# NIR‐Absorbing π‐Extended Azulene: Non‐Alternant Isomer of Terrylene Bisimide

**DOI:** 10.1002/anie.202005376

**Published:** 2020-06-25

**Authors:** Bartłomiej Pigulski, Kazutaka Shoyama, Frank Würthner

**Affiliations:** ^1^ Institut für Organische Chemie Universität Würzburg Am Hubland 97074 Würzburg Germany; ^2^ Center for Nanosystems Chemistry (CNC) Universität Würzburg Theodor-Boveri-Weg 97074 Würzburg Germany

**Keywords:** aromaticity, dyes/pigments, fused-ring systems, structure elucidation, synthetic methods

## Abstract

The first planar π‐extended azulene that retains aromaticity of odd‐membered rings was synthesized by [3+3] *peri*‐annulation of two naphthalene imides at both long‐edge sides of azulene. Using bromination and subsequent nucleophilic substitution by methoxide and morpholine, selective functionalization of the π‐extended azulene was achieved. Whilst these new azulenes can be regarded as isomers of terrylene bisimide they exhibit entirely different properties, which include very narrow optical and electrochemical gaps. DFT, TD‐DFT, as well as nucleus‐independent chemical shift calculations were applied to explain the structural and functional properties of these new π scaffolds. Furthermore, X‐ray crystallography confirmed the planarity of the reported π‐scaffolds and aromaticity of their azulene moiety.

## Graphical abstract

**A non‐alternant** isomer of terrylene bisimide was synthesized by palladium‐catalyzed [3+3] annulation of azulene and naphthalimide. It was functionalized by simple bromination and nucleophilic substitution. These π‐extended azulenes show NIR absorptions and planar geometries with an aromatic azulene moiety, as confirmed by crystallographic analysis and theoretical calculations.

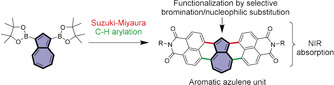

The discovery of fullerenes and carbon nanotubes invigorated synthetic efforts in carbon allotropes and carbon‐rich molecules.^1^ A plethora of benzenoid nanographenes were synthesized by means of synthetic organic chemistry.^2^ However, chemistry of such π‐scaffolds is not restricted to structures containing only hexagons. Incorporation of pentagons, heptagons, or octagons into nanographenes can lead to negative or positive curvatures and different physical properties.^3^ Fully conjugated polycyclic aromatic hydrocarbons (PAHs) are defined as non‐alternant when it is not possible to divide carbon atoms into two groups such that all atoms from the first group have only atoms from the second group as direct neighbors and other way around.^4^ Accordingly, all fully conjugated PAHs containing odd‐membered rings are non‐alternant. Recently, there is a growing interest in such non‐alternant PAHs for organic functional materials,^5^ thereby advancing this traditional field of research beyond fundamental questions on structure and aromaticity.^6^

Among non‐alternant aromatic compounds, azulene is a particularly valued molecule because of its characteristic blue color and unique excited‐state properties. These features motivated chemists to synthesize a variety of azulene‐containing PAHs including nonplanar^7^ and planar^8^ ones by means of solution chemistry as well as more complex structures, such as azulene‐containing graphene nanoribbons by on‐surface synthesis.^9^ PAHs containing such structural motifs show distinctive properties including biradical character,^10^ long‐wavelength absorption,^11^ as well as charge‐transport properties as required for applications in organic transistors^12^ and solar cells.^13^ Fused seven‐ and five‐membered rings of azulene cause different distribution of electron density between HOMO and LUMO. Such non‐centrosymmetric frontier orbital geometry results in an energy gap lower than that for its alternant isomer naphthalene.^14^ However, the unique electronic properties of azulene are usually not preserved when embedded into a larger π‐scaffold. The aromaticity of such azulene moieties is typically lost due to structural distortions.7a, 7b But even if planarity prevails, such PAHs8a, 8b usually possess non‐ or anti‐aromatic “formal azulene“ units because of the dominant aromaticity of the surrounding benzenoid rings. This situation might be overcome by annulation of aromatic units onto the periphery of azulene at positions 1,8 and 3,4, which is, however, challenging because of a lack of synthetic methodologies (Figure 1). To date, indeed most of the reported planar azulenes are *kata*‐annulated on the five‐membered ring.10b, 15 Derivatives with *peri*‐annulation are rare and the small derivative of such annulation, cyclohepta[*def*]fluorene, has been a long‐standing target addressed by many synthetic efforts.^16^

**Figure 1:**
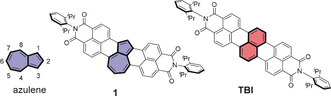
Chemical structures of azulene, the azulene‐containing isomer of **TBI** (**1**), and terrylene bisimide (**TBI**).

Here we report the first planar π‐extended azulene (**1**, Figure 1). In our molecular design two naphthalene building units are annulated to 1,8 and 3,4 positions of azulene to form two new *peri*‐annulated six‐membered rings and preserve the aromaticity of azulene moiety. We took advantage of our synthetic methodology for π‐extended dicarboximides^17^ to synthesize this π‐extended azulene. Notably, the bisimide **1** could be regarded as a new type of non‐alternant isomer of terrylene bisimide (Figure 1). Selective bromination followed by nucleophilic substitution allows further functionalization of the parent compound **1**, which is usually not feasible for other azulene‐containing PAHs.

Synthesis started from the commercially available 1,3‐dibromoazulene (**2**; Scheme 1), which was subjected to Miyaura borylation reaction under modified literature conditions to give the boronic ester **3** in 85 % yield.^18^ Palladium‐catalyzed annulation of **3** with the dibromide **4** using [Pd_2_(dba)_3_⋅CHCl_3_] as a Pd source, PCy_3_⋅HBF_4_ as a ligand, Cs_2_CO_3_ as a base, and a mixture of toluene and water (10:1) as a solvent at 90 °C afforded **1** in 47 % yield. It is noteworthy that our previously reported reaction conditions for the synthesis of **TBI** from the naphthalene analogue of **3** at a higher temperature and with 1‐chloronaphthalene as the solvent17b afforded mainly perylene bisimide (homocoupling product of **4**) and only traces of desired product.

**Scheme 1:**
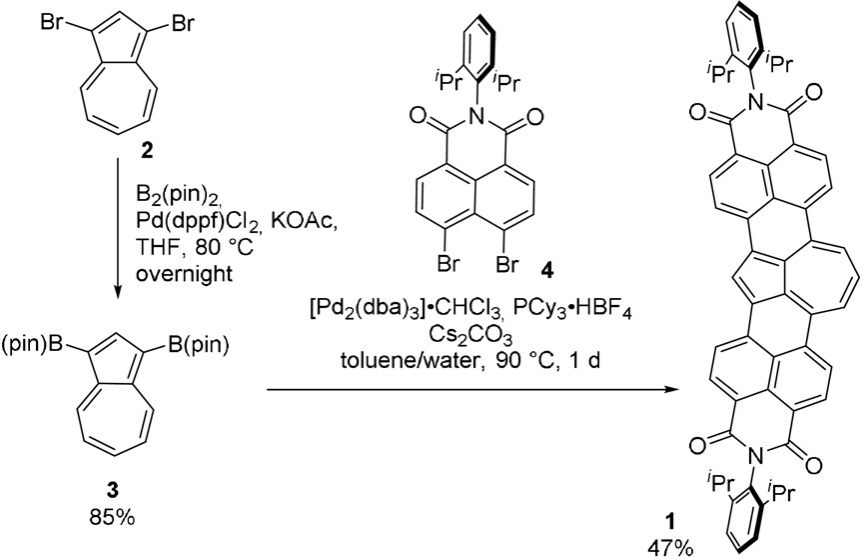
Synthesis of the π‐extended azulene **1**. B_2_pin_2_=bis(pinacolato)diboron, dppf=1,1′‐bis(diphenylphosphino)ferrocene, THF=tetrahydrofuran, B(pin)=boron (pinacol)ester, dba=dibenzylideneacetone, Cy=cyclohexyl.

Mild electrophilic bromination by *N*‐bromosuccinimide (NBS) in tetrahydrofuran leads to a selective substitution at position 2 of azulene, giving the bromide **5** in 80 % yield (Scheme 2). Next, we applied similar synthetic strategy that was used previously for functionalization of naphthalene bisimides by nucleophilic substitution.^19^ Reaction of **5** with an excess of morpholine afforded the amine **6** in 74 % yield. This two‐step functionalization was also possible in a one‐pot protocol directly from **1**. Thus, stirring of **1** with NBS and then addition of morpholine gives **6** in 53 % yield. Synthesis of the methoxy derivative **7** was possible with the use of in situ generated lithium methoxide and the target compound was isolated in 60 % yield.

**Scheme 2:**
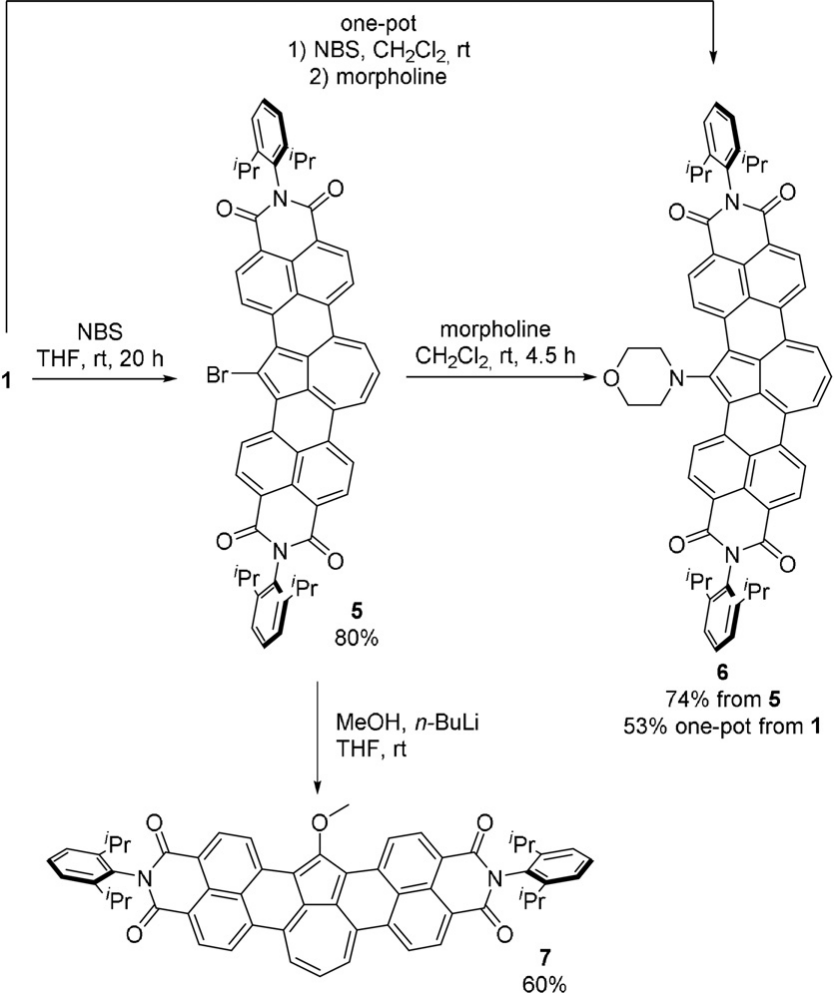
Functionalization of **1**.

Crystallographic analysis was conducted to obtain insight into the structure and aromaticity of π‐extended azulenes. Single crystals of **7** suitable for X‐ray diffraction measurement were grown by slow evaporation of its toluene solution at room temperature.^20^ The crystal structure of **7** (Figure 2) provided unequivocal structural assignment, and solid proof of bromination and further functionalization of parent molecule **1** on position 2 of the azulene moiety.

**Figure 2:**
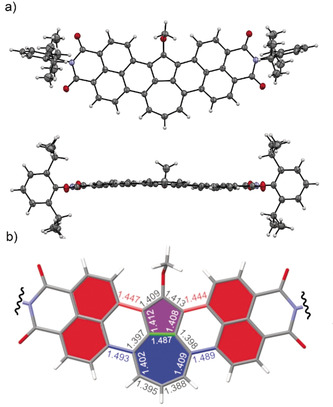
a) ORTEP plot of **7** (top: front view, bottom: side view, ellipsoids set at 50 % probability). b) Visualization of different C−C bond lengths (all values in Å): red rings, benzenoid aromaticity, bond lengths 1.370–1.435 Å, avg. 1.403 Å; violet ring avg. C−C bond length 1.427 Å; blue ring avg. C−C bond length bond lengths 1.410 Å; imide substituents removed for clarity.

The azulene‐embedded π‐scaffold is nearly planar (Figure 2 a) with a mean derivation from an average plane of 0.089 Å. The geometry of azulene unit of **7** is virtually identical to pristine azulene.^21^ Average C−C bond lengths in the five‐membered ring of **7** and azulene are 1.427 Å, whereas those for the seven‐membered ring are very similar (1.410 Å in **7**, 1.405 Å in azulene). The bridging bond between five‐ and seven‐membered rings (Figure 2 b, green bond) is also almost identical in **7** (1.487 Å) and azulene (1.489 Å). The bonds connecting the azulene unit and the naphthalene units are analogous to the *peri*‐bonds in rylenes. However, the bonds between the five‐membered ring and naphthalene units (Figure 2 b, red bonds, 1.444–1.447 Å) are significantly shorter than analogous bonds at the seven‐membered ring (Figure 2 b, blue bonds, 1.489–1.493 Å). The former are significantly shorter than *peri*‐bonds in terrylene bisimide (1.464–1.466 Å),^22^ whereas the latter are comparable to single bonds in biphenyls (1.488 Å).^23^ The bond lengths of naphthalene units (Figure 2 b, red rings) are in a range of values typical for naphthalenes (1.364–1.422 Å, avg. 1.398 Å).^23^ Further analysis of HOMA values based on bond lengths alternation (see Figure S3 in the Supporting Information) confirmed aromaticity of azulene unit of **7**.

To get a deeper insight into the aromaticity of these new π‐scaffolds and in particular of the azulene subunit, we calculated isotropic chemical shielding surface (ICSS)^24^ and nucleus‐independent chemical shifts (NICS)^25^ of **1** (details described in the Supporting Information). An ICSS(1)zz plot (at 1 Å of *Z* axis) for **1** was generated using the Multiwfn package^26^ and it is shown in Figure 3. Red and orange coloring indicate the strongest aromaticity. The two naphthalene units, as well as the five‐membered ring of azulene unit, exhibit accordingly the strongest aromaticity whilst the aromaticity of the seven‐membered ring of **1** is weak. This result is in qualitative agreement with pristine azulene and naphthalene.^27^ NICS(1) values for the five‐membered ring (−10.38) and seven‐membered ring (−1.49) of **1** are in agreement with the ICSS analysis (Figure 3). Likewise, high NICS values are given for the two naphthalene units, whilst the *peri*‐interconnecting rings lack aromaticity, in agreement with the simple bond‐length analysis from the crystallographic data of **7**. Similar NICS(1) values were obtained for the other azulene derivatives, **5**–**7** (see Figures S28–S30), indicating comparable electronic structures. As a most important outcome from ICSS and NICS analyses, the azulene moieties of **1** and **5**–**7** possess intrinsic aromaticity similar to that of the parent azulene, which is usually not the case for π scaffolds containing a “formal azulene” moiety.

**Figure 3:**
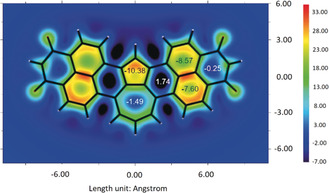
ICSS (isotropic chemical shielding surface) at 1 Å of Z axis (ICSS(1)zz) and NICS(1) values for **1**; Z axis perpendicular to drawing plane, red and orange regions show strong aromaticity values. Imide substituents replaced with hydrogen atoms for simplicity.

The optical properties of π‐extended azulenes were examined by UV/Vis/NIR absorption spectroscopy in dichloromethane solutions at room temperature (Figure 4). The parent compound **1** exhibits resolved vibronic progression for the lowest energy band with *λ*_max_=1041 nm (1.19 eV), which is strongly bathochromically shifted with respect to isomeric terrylene bisimide (*λ*_max_=650 nm, 1.91 eV)^28^ and even hexarylenebisimide (*λ*_max_=953 nm, 1.30 eV).^29^ It is clearly seen that replacing the central naphthalene unit of terrylene with azulene has tremendous impact on optical properties. The substitution at the five‐membered ring as in the derivatives **5**–**7** causes a blueshift of the absorption maxima to *λ*_max_=953 nm (1.30 eV), 921 nm (1.35 eV), and 933 nm (1.33 eV), respectively. Similar to azulene, these π‐extended azulenes show substituent‐dependent colors in solution, and they vary from brown for **1** and **5**, to purple for **6**, and pink for **7** (Figure 4 b).^30^

**Figure 4:**
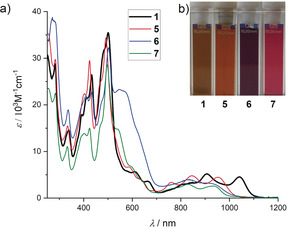
a) UV/Vis/NIR absorption spectra of **1** and **5**–**7** in CH_2_Cl_2_, 298 K, *c*≈10^−5^ m. b) Photographs of **1** and **5**–**7** as CH_2_Cl_2_ solutions under ambient conditions.

DFT calculations provided further details of the optical properties of π‐extended azulenes. HOMO and LUMO plots of **1** and its isomeric terrylene bisimide, as well as pristine azulene are shown in Figure 5. HOMO and LUMO plots for **5**–**7** are similar to **1** and are shown in Figures S10–S15. The azulene moiety of **1** and pristine azulene have essentially the same shape of frontier molecular orbitals, indicating that the original electronic structure of azulene is unaffected in **1**. TD‐DFT calculations proved that the S_0_→S_1_ transitions are mostly HOMO–LUMO transitions and correspond to the lowest absorption band (see Table S4). To analyze the nature of this transition, we calculated the charge‐density difference (CDD) and Δ*r* index.^31^ The former visualizes the increase and decrease of electron density upon excitation and the latter quantifies the distance between the centroids of the hole and electron in the excited state. The CDD plot for the S_0_→S_1_ transition of **1** is visualized in Figure 5 along with that of azulene and **TBI**. Both azulene and **1** exhibit collective shift of electron density alongside the molecular axis from the five‐membered ring to the seven‐membered ring. It is noteworthy that the naphthalene moieties of **1** have electron density displacement in the same direction. Thus, the Δ*r* index for the S_0_→S_1_ transition of **1** (0.698 Å) is only slightly smaller than that of azulene (0.884 Å). In contrast, **TBI** shows equally distributed CDD over the whole molecular scaffold and its Δ*r* index is zero. These data imply that the shift of electron density in the azulene moiety of **1** induced the same shift in its naphthalene moieties. CDD plots and Δ*r* indices for S_0_→S_1_ transition (see Figures S22–S24) of **5**–**7** are comparable to **1**, indicating similar electron‐density shift as **1**. As the result of abovementioned localized hole‐electron distributions of S_0_→S_1_ transition, the repulsion between electrons occupying HOMO and LUMO in the exited state of **1** becomes smaller than that of isomeric **TBI**. This smaller repulsion results in the narrower optical gap of **1** than that of terrylene bisimide (*λ*_max_=1041 nm for **1** and *λ*_max_=650 nm for **TBI** 28), which is akin to pristine azulene and its isomeric naphthalene.^14^

**Figure 5:**
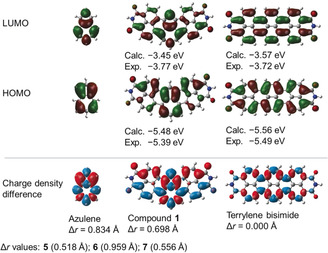
HOMO and LUMO orbitals, and charge density difference (CDD) plots for S_0_→S_1_ transitions. Imide substituents omitted for simplicity, B3LYP/6‐31G(d), isovalue 0.0004 a.u. for HOMO and LUMO; B3LYP/6‐31+G(d,p), isovalue 0.0004 a.u. for CDD. Red (positive) and blue (negative) regions, respectively, represent decreases and increases of electron density after excitation. Experimental HOMO and LUMO levels were taken from electrochemical measurements (see Figures S4–S7 and Table S2).

Interestingly, electrochemically determined frontier orbital energy levels of the reported PAH **1** revealed a wider HOMO–LUMO energy gap (see Figure S7 and Table S2) than corresponding S_0_→S_1_ transitions. This phenomenon is characteristic to azulenes and caused by their non‐alternant nature, which results in localized hole‐electron densities in the excited state and thus a decrease of S_0_→S_1_ transition energy.^14^, 15a Other non‐alternant PAHs also possess significantly lower optical gaps than electrochemical ones.^32^ Thus electrochemically determined HOMO–LUMO energy gap of **1** is 1.62 eV, which is 0.43 eV larger than the excitation energy of the lowest absorption band (1.19 eV, Table S3). This trend is similar in DFT calculations (HOMO–LUMO gap: 2.03 eV, S_0_→S_1_ transition; 1.20 eV: see Table S3). In contrast, PAHs containing only “formal azulene” unit have optical gaps comparable to electrochemical ones,^7^, ^8^ which is an indication for the loss of original electronic properties of pristine azulene.

In summary, we report the bis(dicarboximide) **1**, containing an aromatic azulene unit annulated to two naphthalene imide moieties. Our synthesis was based on a [3+3] annulation consisting of a Suzuki–Miyaura reaction followed by C−H arylation. Bromination of **1** selectively occurred at the remaining free position of the azulene's five‐membered ring, which allowed further functionalization by nucleophilic substitution. The PAH **1** is the first example of π‐extended azulene where positions 1,8 and 3,4 are *peri*‐fused with two naphthalene units at the same time. Such a design preserves the unique electronic properties of the azulene moiety. The aromatic character of the azulene unit and its intrinsic non‐alternant character of frontier molecular orbitals result in a very narrow optical gap and NIR absorption of **1** and all reported derivatives. Such narrow optical gaps usually cannot be reached for neutral, benzenoid PAHs^33^ as well as other azulene‐based dyes.^34^ A crystal structure could be obtained for the methoxy derivative **7**, which corroborated the structural assignment of the series of new azulene molecules and provided information on the aromaticity of these new π scaffolds. Bond‐length analysis indicated that the character of the azulene moiety of the new π‐extended azulenes is similar to that of pristine azulene. This similarity in aromatic character was further supported by ICCS and NICS analyses. To the best of our knowledge this is the first example of a planar π‐extended azulene that retains its original aromatic character.

## Conflict of interest

The authors declare no conflict of interest.

## Supporting information

As a service to our authors and readers, this journal provides supporting information supplied by the authors. Such materials are peer reviewed and may be re‐organized for online delivery, but are not copy‐edited or typeset. Technical support issues arising from supporting information (other than missing files) should be addressed to the authors.

Supplementary
