# Supplementary material for: NIR‐Absorbing π‐Extended Azulene: Non‐Alternant Isomer of Terrylene Bisimide
Source: Angew Chem Int Ed Engl. 2020 Jun 25;59(37):15908–12. doi: 10.1002/anie.202005376 (PMC7540366; doi:10.1002/anie.202005376)
Supplement: Supplementary file 1 — Supplementary [file ANIE-59-15908-s001.pdf]

Angewandte Chemie
Eine Zeitschrift der Gesellschaft Deutscher Chemiker

Supporting Information

NIR-Absorbing π-Extended Azulene: Non-Alternant Isomer of Terrylene Bisimide

*Bartłomiej Pigulski, Kazutaka Shoyama, and Frank Würthner**

anie_202005376_sm_miscellaneous_information.pdf

Table of Contents

Table of Contents S1
1. Methods and Materials S2
2. Synthesis S3
3. X-Ray Crystallography S6
4. Electrochemistry S9
5. DFT Calculations S12
6. NMR Spectra S30
7. Mass Spectra S35
8. References S37

1. Methods and Materials

All reactions dealing with air-or moisture-sensitive compound were carried out by standard Schlenk techniques in dry reaction vessels under nitrogen.

Flash silica-gel column chromatography was performed on silica-gel (particle size 0.040−0.063 mm). Size exclusion column chromatography (SEC) was performed with Bio-Beads S-X3 using dichloromethane and methanol (HPLC grade, 9:1 v/v ratio) as eluent. Recycling gel permeation chromatography (GPC) was performed by a Shimadzu Recycling GPC-System (LC-20AD Prominence Pump; SPDMA20A Prominence Diode Array Detector) with three preparative columns (JAI; JAIGEL) in chloroform (HPLC grade, stabilized with 0.1% EtOH) at 5 mL/min.

Proton nuclear magnetic resonance (^{1}H NMR) and carbon nuclear magnetic resonance (^{13}C NMR) spectra were recorded on a Bruker Avance III HD 400 and Avance III HD 600 spectrometers. Chemical shift data for protons are reported in parts per million (ppm, δ scale) and referenced internally to the residual proton in the solvent ($CDCl_3$: δ 7.26 ppm, C_6D_6: δ 7.16 ppm). Chemical shift data for carbons are reported in parts per million (ppm, δ scale) and referenced internally to the carbon resonance in the solvent ($CDCl_3$: δ 77.16 ppm, C_6D_6, 128.06 ppm). The data are presented as follows: chemical shift, multiplicity (s = singlet, d = doublet, t = triplet, q = quartet, m = multiplet and/or multiple resonances), coupling constant in Hertz (Hz), and integration. Mass spectra were acquired on microTOF focus instrument (Bruker Daltonik GmbH) for high-resolution ESI TOF measurements. Absorption spectra were recorded on JASCO V-770 or -670 spectrometers as dichloromethane (spectroscopy grade) solutions.

Cyclic voltammetry (CV) and square wave voltammetry (SWV) were performed using a standard commercial electrochemical analyser (EC epsilon; BAS Instruments, UK). A Pt disc electrode was used as a working electrode, platinum wire as a counter electrode and Ag^{+}/Ag as a reference electrode, at a scan rate of 100 mV/s at room temperature. Dichloromethane was used as solvent (HPLC grade, purified by SPS) and tetrabutylammonium hexafluorophosphate (0.1 M, recrystallized from ethanol/water and dried under vacuum) as supporting electrolyte. The oxidation potentials were referenced against the ferrocenium/ferrocene redox couple.

Theoretical calculations were performed by Gaussian software[1] using B3LYP/6-31G(d) level theory for structure optimization, B3LYP/6-31+G(d,p) level theory for TD-DFT simulation of electron transitions and B3LYP/6-31+G(d,p) level theory for ICSS mapping and NICS calculations. Multifwn software was used for analysis of electron transitions, charge density difference plots, ICSS mapping and HOMA values calculations.[2] Optimized ground state geometries were examined by frequency analysis to possess no negative frequency.

Solvents for synthesis and silica-gel chromatography were treated as follows: THF (HPLC grade, purified by SPS); MeOH (dried with the use of molecular sieves, 4 Å); toluene, acetone, hexane and dichloromethane were used as received. Following reagents were purified as follows: $B_2(pin)_2$ (sublimed before use), KOAc (dried before use under vacuum) and PCy_3·HBF_4 (recrystallized from ethanol). Following reagents were used without additional purification: Pd(dppf)Cl_2, Cs_2CO_3, *n*-BuLi (1.6 M in hexanes), NBS, morpholine. 1,3-Dibromoazulene **2**,[3] dibromoimide **4**,[4] and [$Pd_2(dba)_3$]·$CHCl_3$[5] were prepared using literature procedures.

The diffraction images for X-ray crystallographic analysis were collected on a Bruker D8 Quest Kappa diffractometer with a PhotonII detector and multi-layered mirror monochromated Cu Kα radiation. The positional and thermal parameters were refined by the full-matrix least-squares method using SHELXL-2018/3 program.[6]

2. Synthesis

1,3-Bis(4,4,5,5-tetramethyl-1,3,2-dioxaborolan-2-yl)azulene (3)

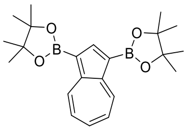

1,3-Dibromoazulene (0.200 g, 0.699 mmol) was placed in a Schlenk flask (100 mL). Then Pd(dppf)Cl_2 (0.102 g, 0.140 mmol), dry KOAc (0.412 g, 4.20 mmol) and dry B_2(pin)$_2$ (0.622 g, 2.45 mmol) were added to the flask. Afterwards, dry THF was added and the reaction mixture was purged with N_2. The resulting mixture was stirred overnight at 80 °C under N_2 in sealed flask. After removing solvent under reduced pressure, the resulting mixture was dissolved in a small amount of CH_2Cl_2 and then filtrated. The crude product was purified by silica-gel chromatography (CH_2Cl_2) to afford a violet solid. Yield 0.226 g (0.595 mmol), 85%.
^{1}H NMR (400 MHz, $CDCl_3$) δ 9.10 (d, *J* = 10.0 Hz, 2H), 8.76 (s, 1H), 7.65 (t, *J* = 9.8 Hz, 1H), 7.40 (t, *J* = 10.0, Hz, 2H).
^{1}H NMR spectrum was consistent with the literature data.[7]

N,*N′*-Bis(2,6-diisopropylphenyl)diphenaleno[1,2,3-*cd*:1′,2′,3′-*ij*]azulene-3,4:12,13-bis(dicarboximide) (1)

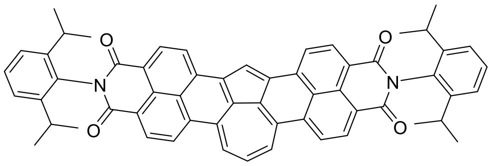

Eight parallel reactions were carried out: 1,3-Bis(4,4,5,5-tetramethyl-1,3,2-dioxaborolan-2-yl)azulene (**3**, 0.0100 g, 0.026 mmol), *N*-(2,6-diisopropylphenyl)-4,5-dibromonaphthalene-1,8-dicarboximide (**4**, 0.0298 g, 0.058 mmol), $Pd_2(dba)_3 \cdot CHCl_3$ (0.0054 g, 0.005 mmol), $PCy_3 \cdot HBF_4$ (0.0039 g, 0.011 mmol), Cs_2CO_3 (0.0771 g, 0.237 mmol), 4 mL of toluene and 0.4 mL of water were placed in a 15 mL borosilicate glass screw-sealed vial (PTFE liner, solid cap). Each vial was evacuated and filled with N_2 (three cycles) and then carefully sealed. All vials were placed in pre-heated heating block and stirred for 1 day at 90 °C. All reaction mixtures were combined and then solvent was removed under reduced pressure. First, mixture was purified with the use of flash silica-gel chromatography (CH_2Cl_2, dark brown fraction). Finally, the product was purified with the use of size exclusion chromatography (Bio-Beads S-X3, DCM/methanol; v/v; 9/1). Dark brown solid (0.0811 g, 0.097 mmol), yield: 47%. Mp: 296.1–307.1 °C (decomposition)
^{1}H NMR (400 MHz, $CDCl_3$) δ = 8.64 (d, *J* = 8.2, 2H), 8.61 (s, 1H), 8.59 (d, *J* = 7.9, 2H), 8.53 (d, *J* = 8.2, 2H), 8.12 (d, *J* = 7.9, 2H), 7.97 (d, *J* = 10.9, 2H), 7.58 (t, *J* = 10.9, 1H), 7.50 (t, J = 7.9, 1H), 7.35 (d, *J* = 7.8, 2H), 2.75 (hept, *J* = 6.7, 4H), 1.19 (dd, *J* = 6.7, 3.5, 24H).
^{13}C NMR (101 MHz, $CDCl_3$) δ = 163.8, 163.7, 145.7, 142.7, 138.9, 138.8, 137.2, 136.3, 133.2, 131.2, 130.9, 130.6, 129.7, 127.8, 127.1, 126.6, 125.4, 124.8, 124.4, 124.2, 120.8, 119.6, 29.3, 24.2.
UV/Vis/NIR (CH_2Cl_2): λ_{max}, nm (ε, M^{-1} cm^{-1}): 1041 (4500), 908 (5200), 666 (3600), 612 (5600), 612 (5600), 501 (35500), 433 (26400).
ESI-HRMS (positive mode) m/z calcd for $C_{58}H_{46}N_2NaO_4^+$ (M+Na$^+$): 857.33498; found: 857.33449.

N,*N′*-Bis(2,6-diisopropylphenyl)-16-bromodiphenaleno[1,2,3-*cd*:1′,2′,3′-*ij*]azulene-3,4:12,13-bis(dicarboximide) (5)

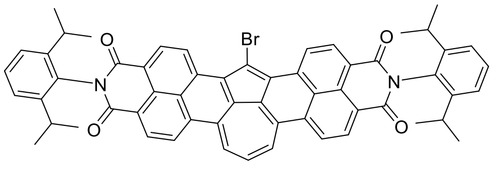

Compound **1** (0.045 g, 0.054 mmol) was dissolved in dry THF (50 mL) and NBS (0.0192 g, 0.108 mmol) was added. The reaction mixture was stirred for 20 h at room temperature. Afterwards, 100 mL of Et_2O was added to the reaction mixture. The organic solution was washed twice with water (2 × 50 mL), twice with saturated aqueous solution of K_2CO_3 (2 × 50mL) and with water again (2 × 50 mL). The combined organic layers were dried with $MgSO_4$, filtrated and then solvent was removed under reduced pressure. The crude product was purified by size exclusion chromatography (BioBeads S-X3; CH_2Cl_2/MeOH; v/v; 9/1) and isolated as brown solid, yield: 80% (0.0393 g, 0.043 mmol). Mp: >350 °C

^{1}H NMR (400 MHz, $CDCl_3$) δ 9.43 (d, *J* = 8.2 Hz, 2H), 8.69 (d, *J* = 8.1 Hz, 2H), 8.63 (d, *J* = 8.2 Hz, 2H), 8.55 (d, *J* = 8.3 Hz, 2H), 8.00 (d, *J* = 11.0 Hz, 2H), 7.59 (t, *J* = 10.6 Hz, 1H), 7.51 (m, 2H), 7.37 (d, *J* = 7.8 Hz, 4H), 2.78 (hept, *J* = 6.8 Hz, 4H), 1.21 (dd, *J* = 6.8, 3.8 Hz, 24H).

^{13}C NMR (151 MHz, $CDCl_3$) δ 163.9, 163.7, 145.8, 141.2, 138.3, 137.1, 136.7, 135.5, 133.0, 130.9, 130.8, 130.2, 129.8, 128.4, 126.7, 125.2, 124.4, 124.2, 124.1, 123.2, 121.6, 120.0, 29.4, 24.2.

UV/Vis/NIR (CH_2Cl_2): λ_{max}, nm (ε, M^{-1} cm^{-1}): 953 (4500), 848 (4800), 7600 (3400), 586 (6700), 492 (34400), 424 (29500).

ESI-HRMS (positive mode) m/z calcd for $C_{58}H_{45}BrN_2NaO_4^+$ (M+Na$^+$): 935.24549; found: 935.24398.

N,*N′*-Bis(2,6-diisopropylphenyl)-16-(morpholin-4-yl)diphenaleno[1,2,3-*cd*:1′,2′,3′-*ij*]azulene-3,4:12,13-bis(dicarboximide) (6)

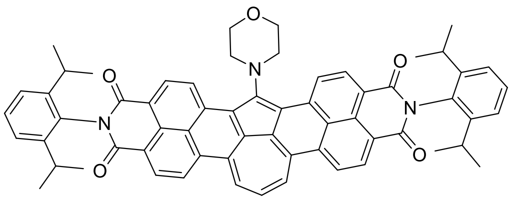

*From compound **5***

Compound **5** (0.0062 g, 0.0068 mmol) was dissolved in CH_2Cl_2 (20 mL) and morpholine (5.9 µL, 0.068 mmol) was added dropwise. The reaction mixture was stirred at room temperature for 4.5 h and solvent was removed under reduced pressure. The product was purified by silica-gel chromatography (CH_2Cl_2, next CH_2Cl_2/acetone; v/v; 95/5), yielding 0.0046 g (0.0050 mmol) of violet solid. Yield: 74%

*From compound **1***

Compound **1** (0.0050 g, 0.0060 mmol) was dissolved in CH_2Cl_2 (20 mL) and NBS (0.0021 g, 0.012 mmol) was added. The mixture was stirred for 3.5 h and then morpholine (5.2 µL, 0.060 mmol) was added. The mixture was stirred for an additional 17 h and the solvent was removed under reduced pressure. The crude product was purified using silica-gel column (CH_2Cl_2, next CH_2Cl_2/acetone; v/v; 95/5) yielding 0.0029 g (0.0032 mmol) of **6** as a violet solid. Yield: 53%. Mp: 237.6–240.1 °C

^{1}H NMR (400 MHz, C_6D_6) δ 8.78 (d, *J* = 8.0 Hz, 2H), 8.73 (d, *J* = 8.0 Hz, 2H), 7.91 (d, *J* = 8.0 Hz, 2H), 7.79 (d, *J* = 8.3 Hz, 2H), 7.37 (dd, *J* = 8.6, 6.9 Hz, 2H), 7.29 (m, 4H), 7.27 (d, *J* = 10.9 Hz, 2H), 6.87 (t, *J* = 10.7 Hz, 1H), 3.68 (t, *J* = 4.7 Hz, 4H), 3.12 (hept, *J* = 6.9 Hz, 4H), 2.87 (t, *J* = 4.7 Hz, 4H), 1.30 (dd, *J* = 6.8, 1.1 Hz, 24H).

^{13}C NMR (101 MHz, C_6D_6) δ 164.1, 163.9, 156.6, 146.4, 139.5, 138.7, 137.6, 135.6, 132.8, 132.1, 130.7, 130.5, 129.8, 129.2, 128.7, 127.4, 127.0, 124.4, 124.2, 124.0, 122.4, 66.5, 46.7, 29.9, 24.3, 24.3.

UV/Vis/NIR (CH_2Cl_2): λ_{max}, nm (ε, M^{-1} cm^{-1}): 921 (3100), 828 (4000), 545 (23300), 502 (32300), 434 (22800), 409 (22000).

ESI-HRMS (positive mode) m/z calcd for $C_{62}H_{53}N_3NaO_5^+$ (M+Na$^+$): 942.38774; found: 942.38636.

***N*,*N′*-Bis(2,6-diisopropylphenyl)-16-methoxydiphenaleno[1,2,3-*cd*:1′,2′,3′-*ij*]azulene-3,4:12,13-bis(dicarboximide) (7)**

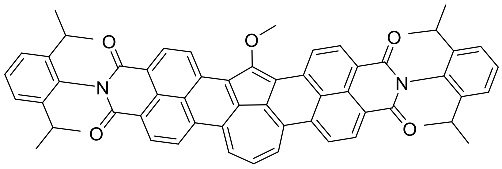

Compound **5** (0.0080 g, 0.0087 mmol) was placed in a Schlenk flask and dissolved in dry THF (3 mL) and dry MeOH (0.1 mL). Afterwards *n*-BuLi (1.6 M in hexanes, 55 µL, 0.088 mmol) was added dropwise and the mixture was stirred for 40 min at room temperature under N_2. Then the reaction was carefully quenched with a few drops of water and passed through a short Al_2O_3 plug (neutral, Brockmann grade I, CH_2Cl_2, next CH_2Cl_2/MeOH, v/v, 95/5). The crude product was purified with the use of silica-gel chromatography (CH_2Cl_2) and then GPC ($CHCl_3$), yielding a dark pink solid, yield 60% (0.0045 g, 0.0052 mmol). Mp: 290.2–291.3 °C

^{1}H NMR (400 MHz, $CDCl_3$) δ 8.67 (m, 4H), 8.58 (m, 4H), 8.14 (d, *J* = 10.8 Hz, 2H), 7.65 (t, *J* = 10.8, 1H), 7.49 (d, *J* = 7.8 Hz, 2H), 7.35 (d, *J* = 7.8 Hz, 4H), 4.17 (s, 3H), 2.76 (hept, *J* = 6.9 Hz, 4H), 1.19 (dd, *J* = 6.8, 2.9 Hz, 24H).

^{13}C NMR (101 MHz, $CDCl_3$) δ 165.5, 163.9, 163.8, 145.8, 141.2, 137.6, 137.0, 136.8, 135.5, 133.7, 131.1, 131.0, 130.4, 129.7, 128.1, 126.3, 124.3, 124.2, 123.9, 121.6, 119.2, 116.8, 58.3, 29.3, 24.2.

UV/Vis/NIR (CH_2Cl_2): λ_{max}, nm (ε, M^{-1} cm^{-1}): 933 (2600), 829 (3000), 528 (14800), 497 (28600), 424 (15000).

ESI-HRMS (positive mode): m/z calcd for $C_{59}H_{48}N_2NaO_5^+$ (M+Na$^+$): 887.34554; found: 887.34452.

3. X-Ray Crystallography

Table S1. Details of X-ray single crystal experiment for compound **7** (CCDC 1996228).

Crystal Data	
Chemical formula	$2(C_{59}H_{48}N_2O_5)\cdot 3(C_7H_8)$
M_r	2006.38
Crystal system, space group	Triclinic, $P\bar{1}$
Temperature (K)	100
a, *b*, *c* (Å)	9.7701 (4), 15.7471 (7), 17.9856 (8)
α, β, γ (°)	98.225 (2), 95.193 (2), 104.217 (2)
V (Å^3)	2631.9 (2)
Z	1
Radiation type	Cu *K*α
μ (mm^{-1})	0.62
Crystal size (mm)	0.21 × 0.20 × 0.14
Data collection	
Diffractometer	Bruker-Kappa-D8Quest_PhotonII
Absorption correction	Multi-scan<br>Bruker-*SADABS*
T_{min}, T_{max}	0.635, 0.754
No. of measured reflections	40288
No. of independent reflections	10353
No. of observed [$I > 2\sigma(I)$] reflections	8344
R_{int}	0.042
$(\sin \theta/\lambda)_{max}$ (Å^{-1})	0.619
Refinement	
$R[F^2 > 2\sigma(F^2)]$, $wR(F^2)$, S	0.061, 0.171, 1.04
No. of reflections	10353
No. of parameters	720
No. of restrains	1
H-atom treatment	H-atom parameters constrained
$\Delta\rho_{max}$, $\Delta\rho_{min}$ (e Å^{-3})	0.41, −0.27

Computer programs: Bruker *APEX3*, Bruker *SAINT*, SHELXT 2014/5 (Sheldrick, 2014), *SHELXL2018*/3 (Sheldrick, 2018).

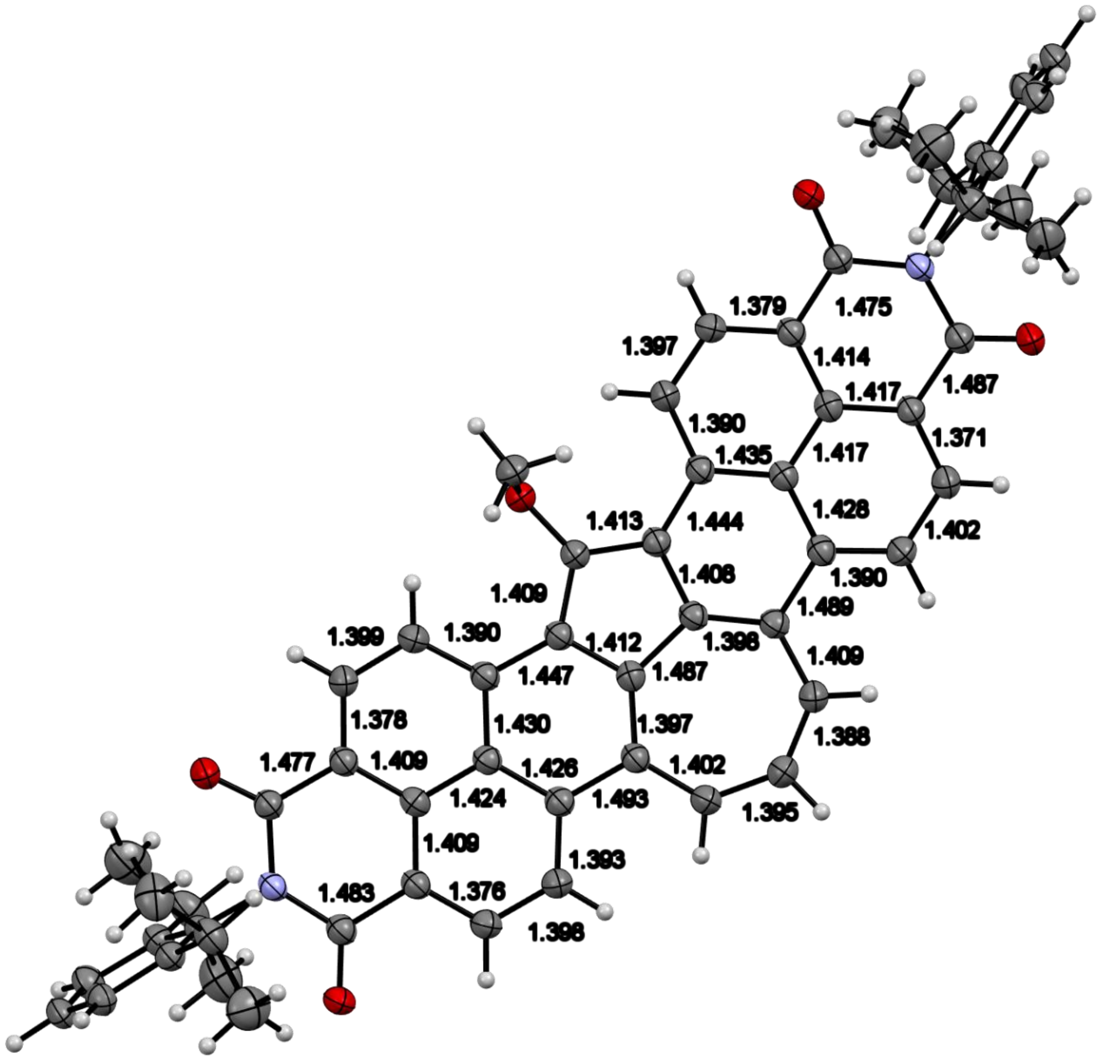

Figure S1. Selected C-C bond lengths of **7** determined by crystallographic analysis (Å).

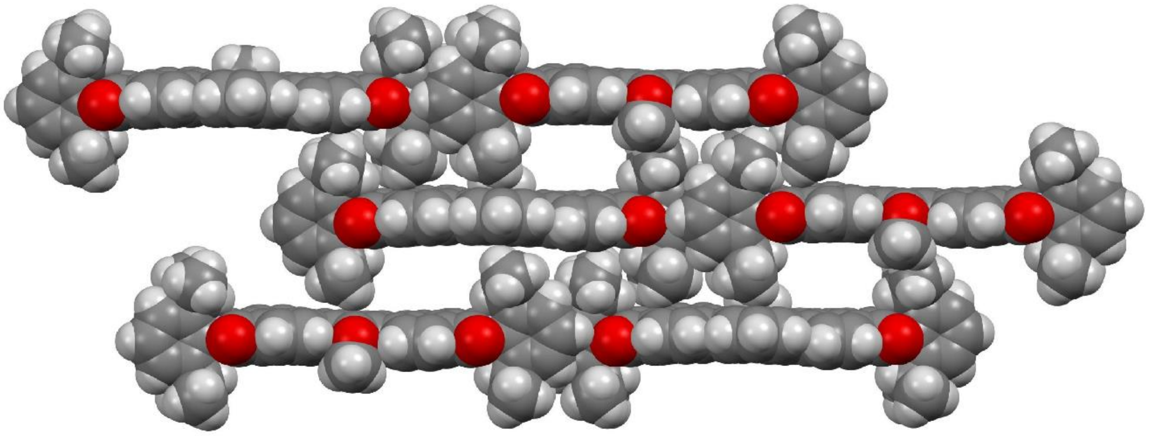

Figure S2. Packing of **7** with toluene molecules removed for clarity.

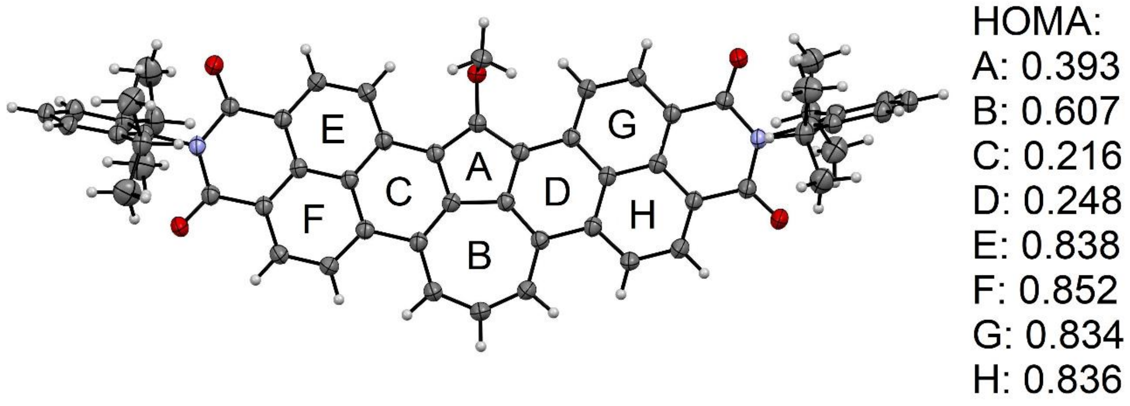

Figure S3. HOMA values calculated for 7 using the structure obtained by X-ray crystallography.

HOMA values analysis

Bond lengths aromaticity of **7** could be analyzed by the harmonic oscillator model of aromaticity (HOMA).[8] In an ideal case, the HOMA value is 1.0 when there is no bond-length alternation in an aromatic system. HOMA values for the four six-membered rings of the two naphthalene units (rings E–H, 0.838–0.852) indicate strong, benzenoid aromaticity, whereas rings C and D are weakly aromatic (HOMA values 0.216 and 0.248, respectively). This result resembles strongly the one for rylene bisimides. HOMA values for azulene moiety are relatively low: 0.393 for five- and 0.607 for seven-membered ring, respectively. However, since HOMA index is based on bond-length alternation and works better for benzenoid structures, values for compound **7** were compared with those calculated for pristine azulene. The HOMA values for unsubstituted azulene based on literature X-ray structure[9] are 0.310 for five-membered ring and 0.544 for seven-membered ring. This indicates that the bond-length alternation of the azulene moiety of **7** is comparable with that of pristine azulene.

4. Electrochemistry

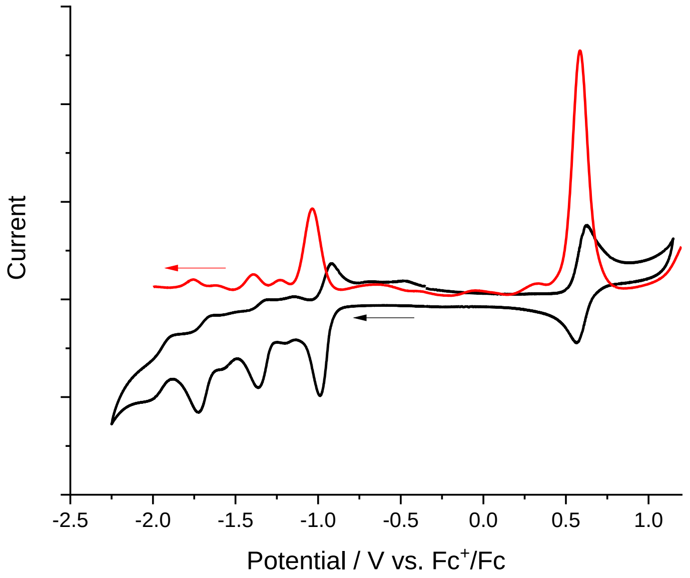

Figure S4. Cyclic (black) and square wave (red) voltammogram of compound **1** (298 K, CH_2Cl_2).

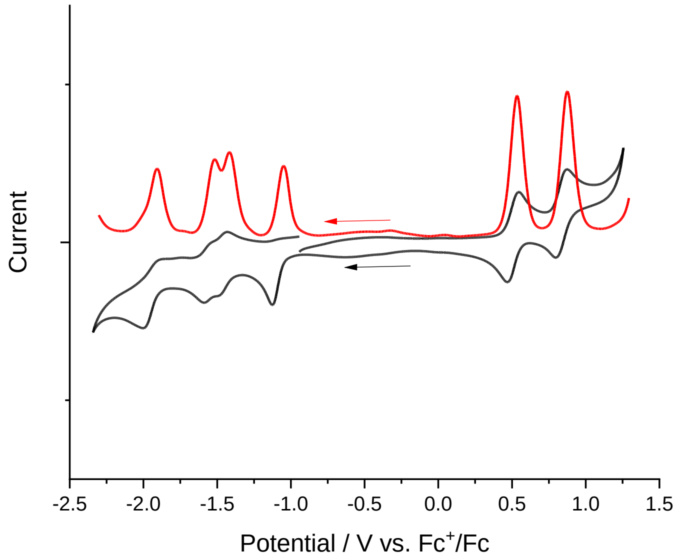

Figure S5. Cyclic (black) and square wave (red) voltammogram of compound **6** (298 K, CH_2Cl_2).

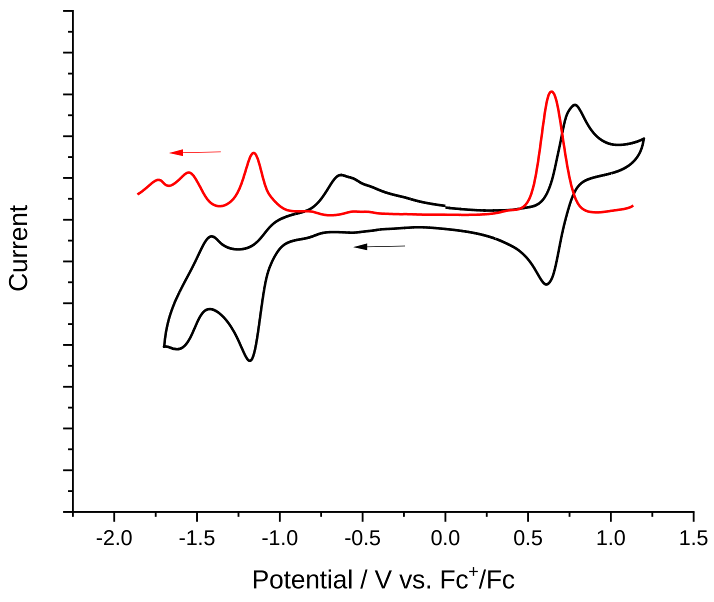

Figure S6. Cyclic (black) and square wave (red) voltammogram of compound **7** (298 K, CH_2Cl_2).

Table S2. Oxidation and reduction potentials for compounds **1** and **6**–**7**. Potentials taken from square wave voltammetry measurements (SWV), CH_2Cl_2, room temperature.

Compound	**1**	**5**[a]	**6**	**7**
Reduction [V vs. Fc^+/Fc]	–1.03	-	–1.14	–1.11
Oxidation [V vs. Fc^+/Fc]	0.59	-	0.53, 0.88	0.64
HOMO energy (exp.)[b] [eV]	–5.39	-	–5.33	–5.44
LUMO energy (exp.)[b] [eV]	–3.77	-	–3.66	–3.69
Gap [eV]	1.62	-	1.67	1.75

[a] Reliable electrochemical data could not be obtained due to poor solubility. [b] Calculated using Fc^+/Fc redox couple (–4.8 eV) as reference.

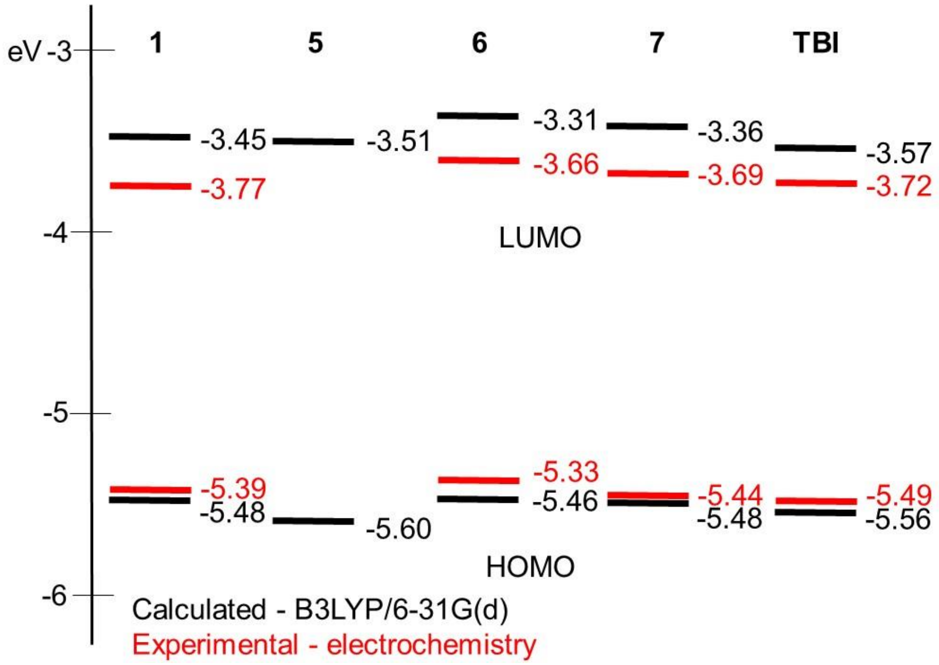

Figure S7. Experimental HOMO and LUMO energy levels (red) determined from electrochemistry (calculated using Fc^{+}/Fc redox couple (–4.8 eV) as a reference) and DFT calculations (black, B3LYP/6-31G(d)). Experimental data for **TBI** was taken from the literature[10] and calibrated with respect to Fc^{+}/Fc redox couple.[11]

5. DFT Calculations

HOMO and LUMO orbitals; Imide substituents replaced with hydrogen atoms for simplicity. B3LYP/6-31G(d), isovalue 0.0004 a.u.

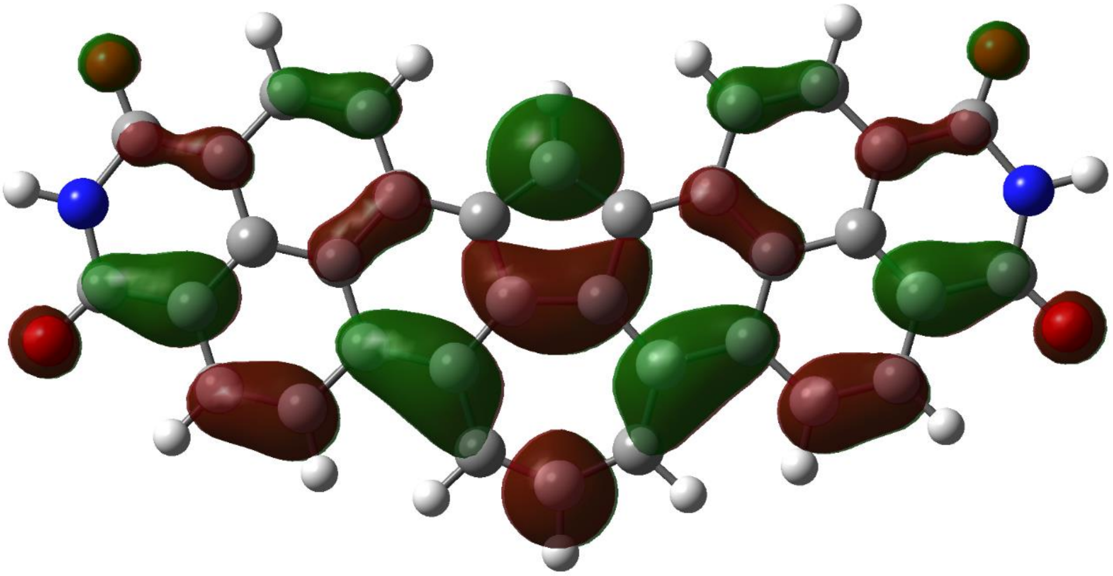

Figure S8. LUMO plot of compound **1**.

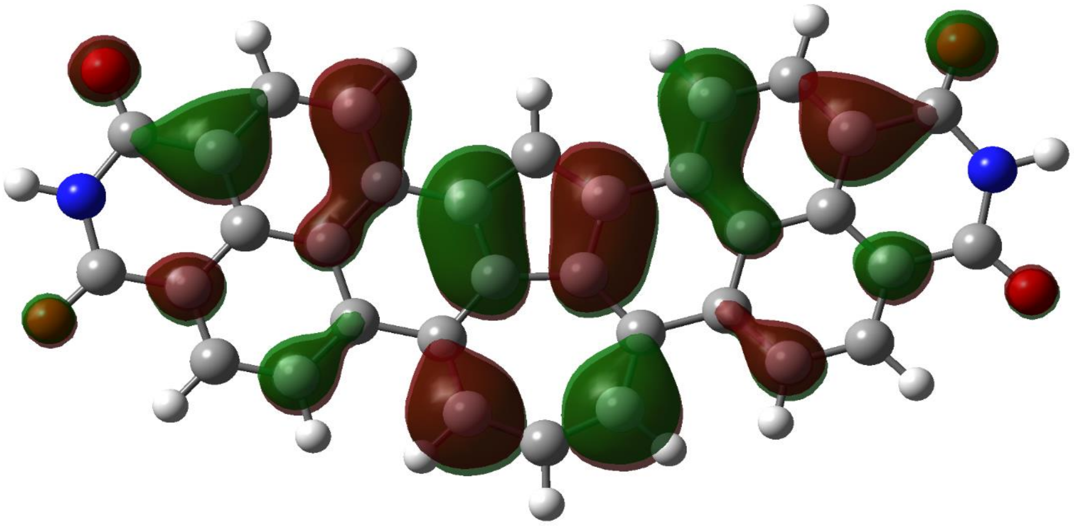

Figure S9. HOMO plot of compound **1**.

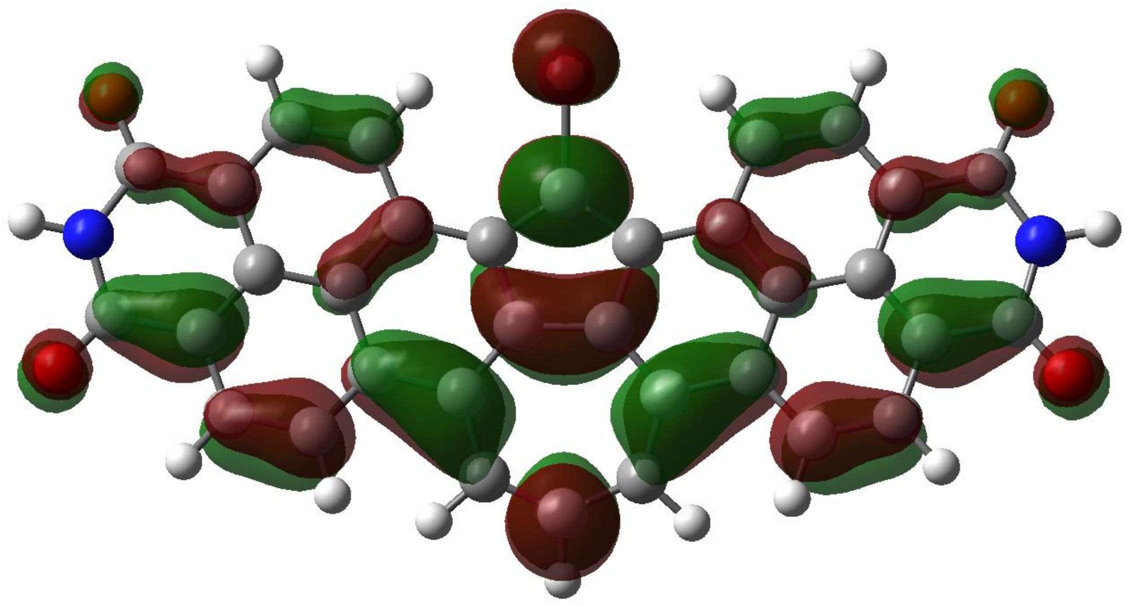

Figure S10. LUMO plot of compound **5**.

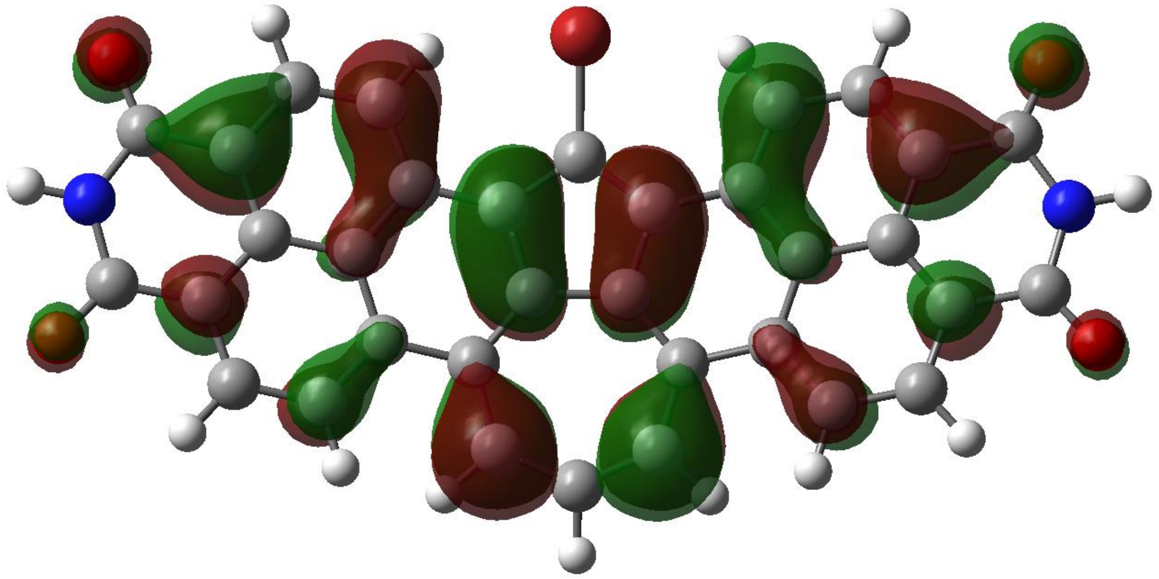

Figure S11. HOMO plot of compound **5**.

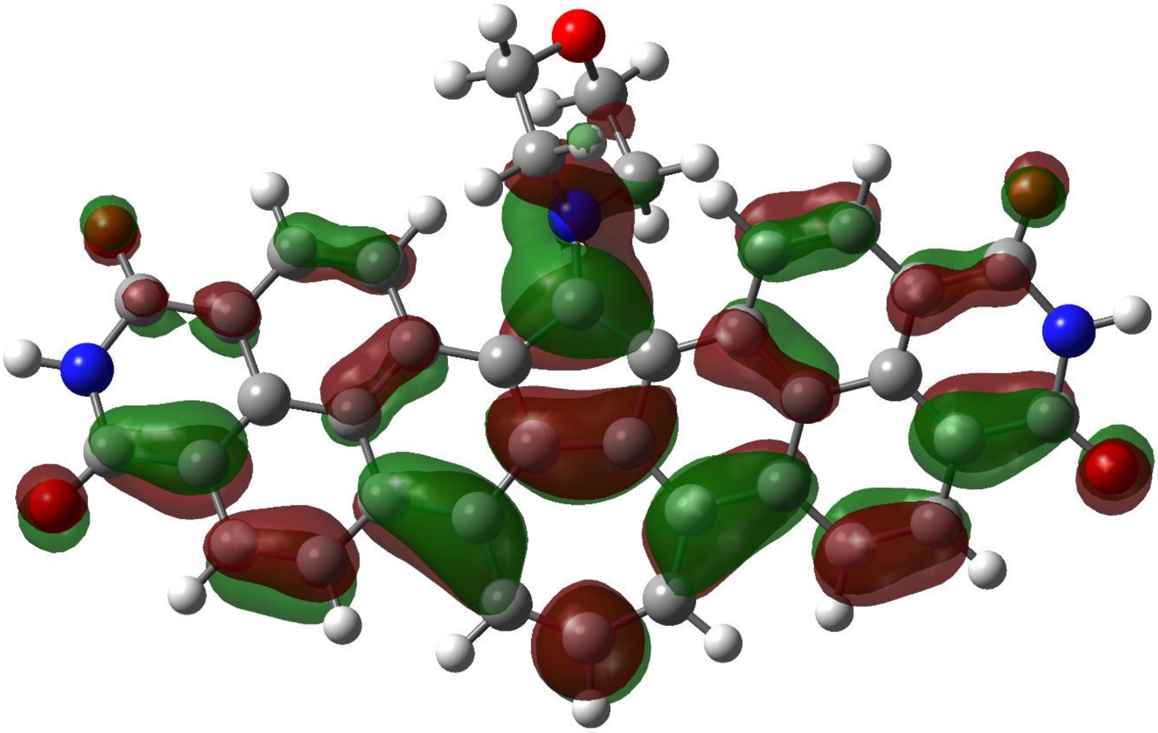

Figure S12. LUMO plot of compound **6**.

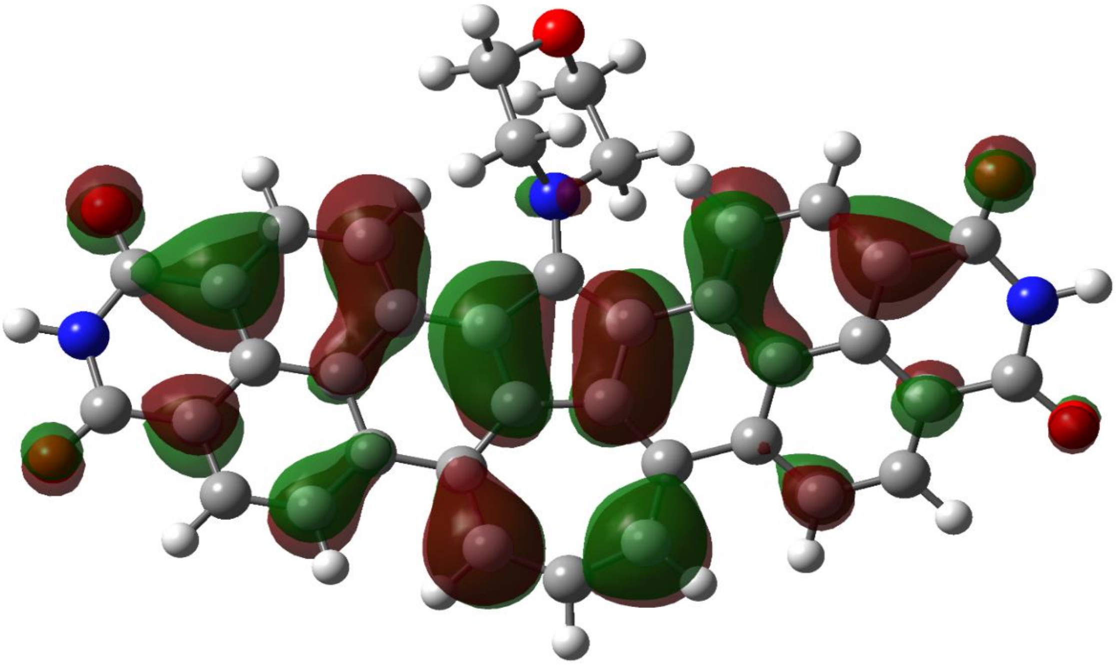

Figure S13. HOMO plot of compound **6**.

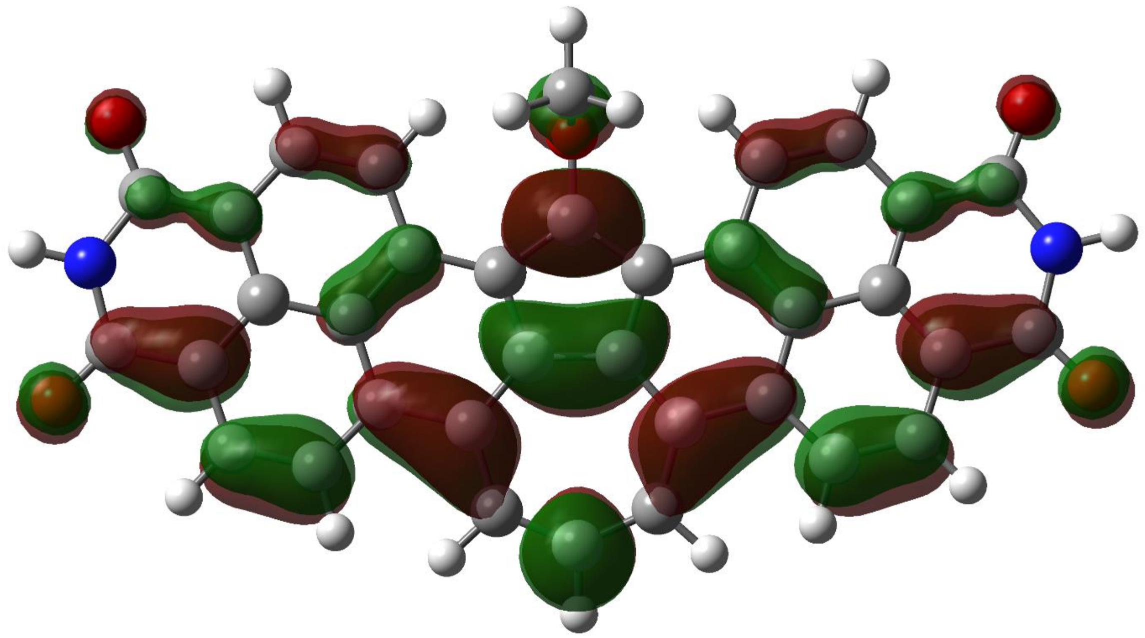

Figure S14. LUMO plot of compound **7**.

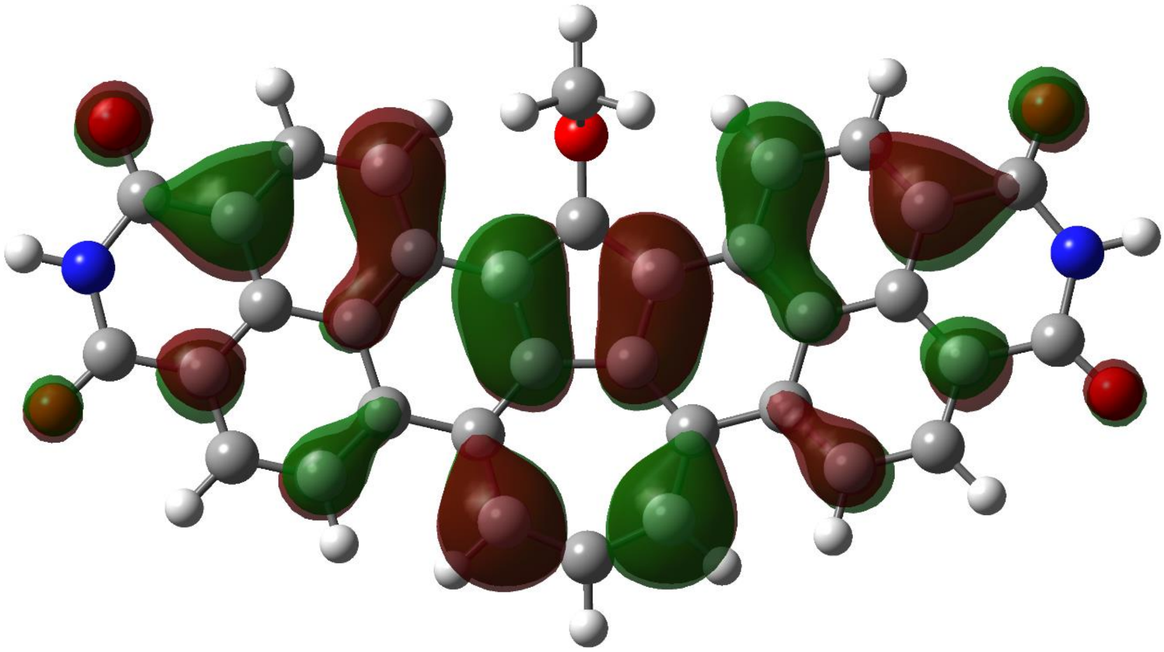

Figure S15. HOMO plot of compound **7**.

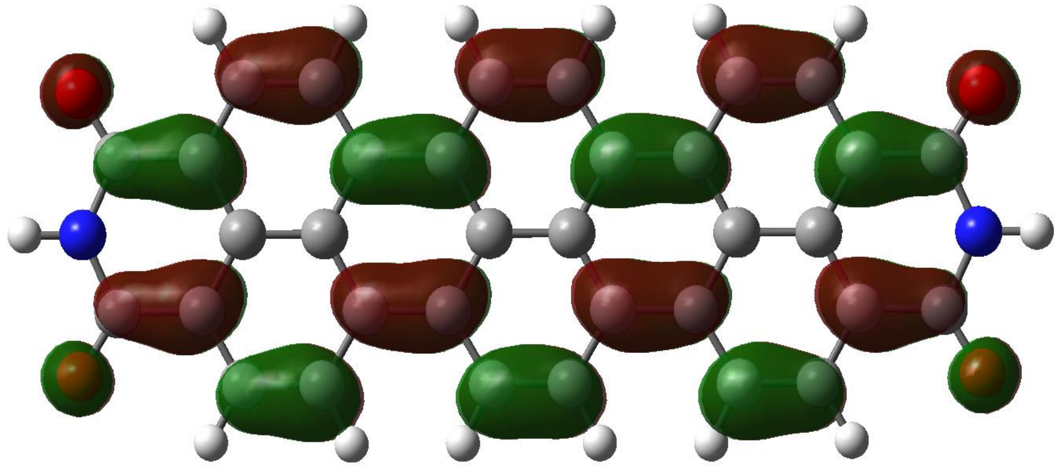

Figure S16. LUMO plot of **TBI**.

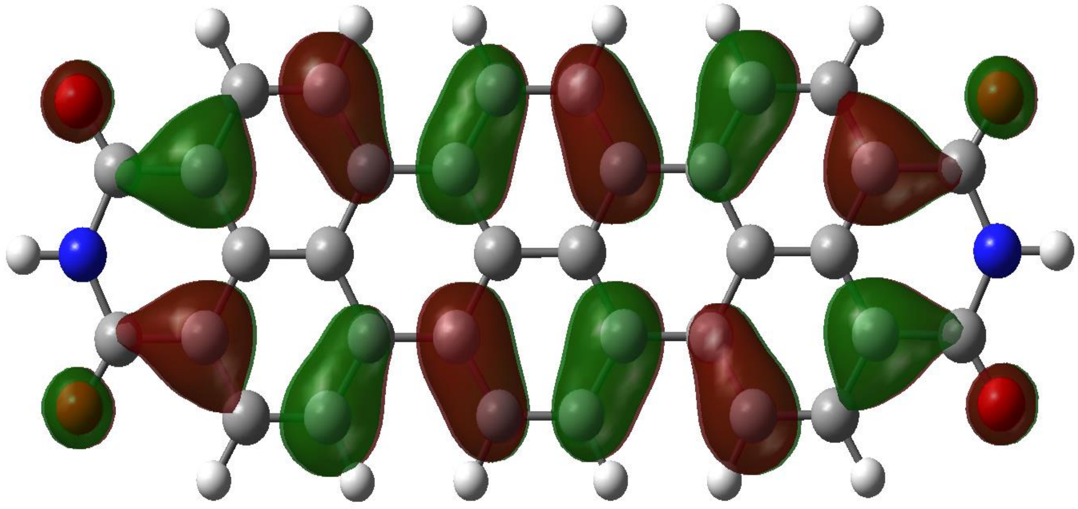

Figure S17. HOMO plot of **TBI**.

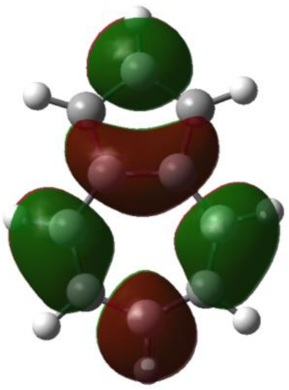

Figure S18. LUMO plot of azulene.

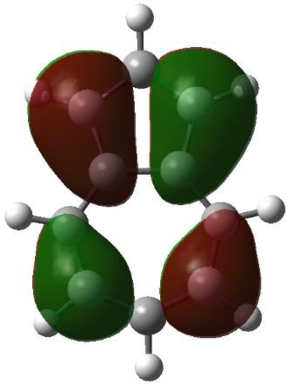

Figure S19. HOMO plot of azulene.

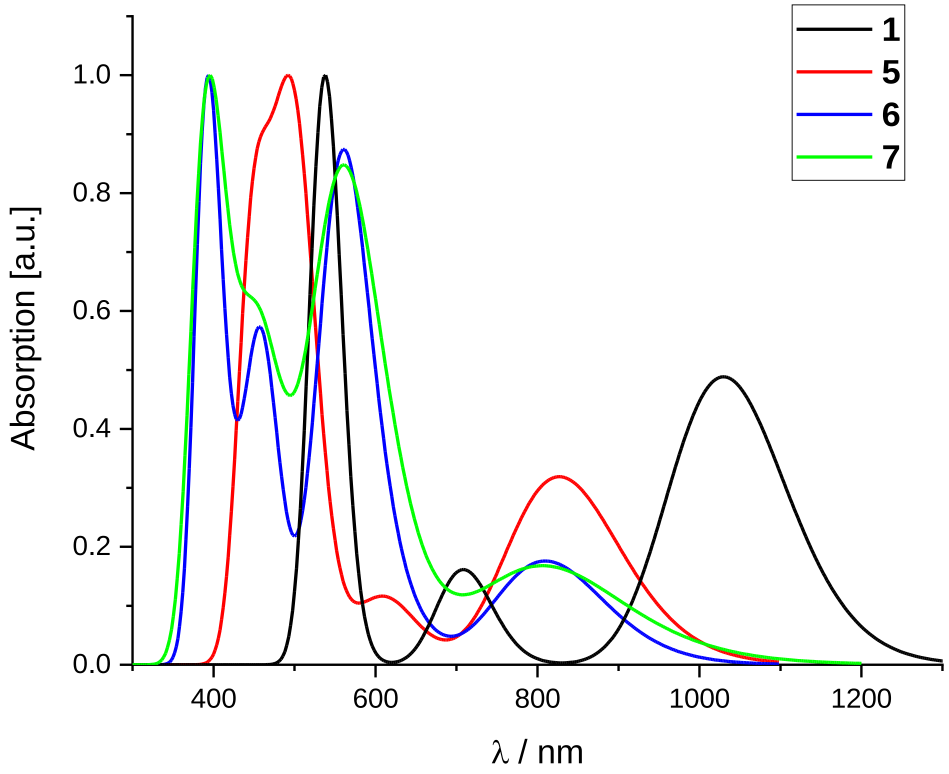

Figure S20. Simulated absorption spectra for **1**, **5**–**7**.

Table S3. Maxima of the lowest energy absorption bands (HOMO–LUMO): experimental and calculated using TD-DFT (B3LYP/6-31+G(d,p)).

Compound	λ_{max} (exp.)	Energy (exp.)	λ_{max} (calc.)	Energy (calc.)
**1**	1041 nm	1.19 eV	1030 nm	1.20 eV
**5**	953 nm	1.30 eV	833 nm	1.49 eV
**6**	931 nm	1.33 eV	810 nm	1.53 eV
**7**	932 nm	1.33 eV	820 nm	1.51 eV
**TBI**	650 nm[12]	1.91 eV	572 nm	2.17 eV
azulene	579 nm[13]	2.14 eV	519 nm	2.39 eV

Table S4. Contributions to electron transitions (B3LYP/6-31+G(d,p)).

Transition	Main contribution	Energy [eV]	Wavelength [nm]	Oscillator strength
Compound **1**				
S_1	HOMO→LUMO (0.695)	1.20	1033	0.127
S_2	HOMO→LUMO+1 (0.687)	1.75	708	0.042
S_3	HOMO-1→LUMO (0.682)	2.21	561	0.002
S_4	HOMO-1→LUMO+1 (0.690)	2.31	537	0.259
Compound **5**				
S_1	HOMO→LUMO (0.691)	1.49	832	0.123
S_2	HOMO→LUMO+1 (0.693)	2.04	608	0.047
S_3	HOMO-1→LUMO (0.691)	2.39	519	0.009
S_4	HOMO-1→LUMO+1 (0.690)	2.47	502	0.360
Compound **6**				
S_1	HOMO→LUMO (0.685)	1.53	810	0.088
S_2	HOMO→LUMO+1 (0.691)	1.99	623	0.039
S_3	HOMO-1→LUMO (0.539)<br>HOMO-1→LUMO+1 (0.429)	2.20	564	0.142
S_4	HOMO-1→LUMO (-0.436)<br>HOMO-1→LUMO+1 (0.539)	2.22	558	0.287
Compound **7**				
S_1	HOMO→LUMO (0.690)	1.51	821	0.104
S_2	HOMO→LUMO+1 (0.695)	2.00	620	0.053
S_3	HOMO-1→LUMO (0.690)	2.39	519	0.003
S_4	HOMO-1→LUMO+1 (0.690)	2.41	514	0.407
Azulene				
S_1	HOMO→LUMO (0.699)	2.39	519	0.007
S_2	HOMO→LUMO+1 (0.518)<br>HOMO-1→LUMO (0.479)	3.61	343	0.003
S_3	HOMO-1→LUMO+1 (0.641)<br>HOMO-2→LUMO (0.268)	4.63	268	0.055
S_4	HOMO-1→LUMO+2 (0.702)	4.78	259	0.000

Charge density difference plots

Imide substituents replaced with hydrogen atoms for simplicity. (B3LYP/6-31+G(d,p), isovalue 0.0004 a.u.). Red (positive) and blue (negative) regions represent decreases and increases, respectively, of electron density after excitation.

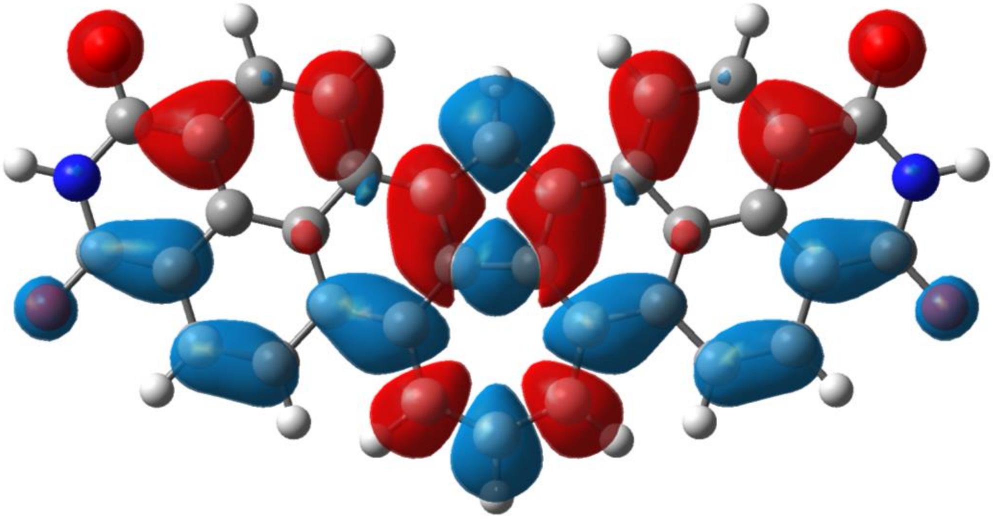

Figure S21. Charge density difference for $S_0 \longrightarrow S_1$ transition of compound **1**; Δ*r* = 0.698 Å.

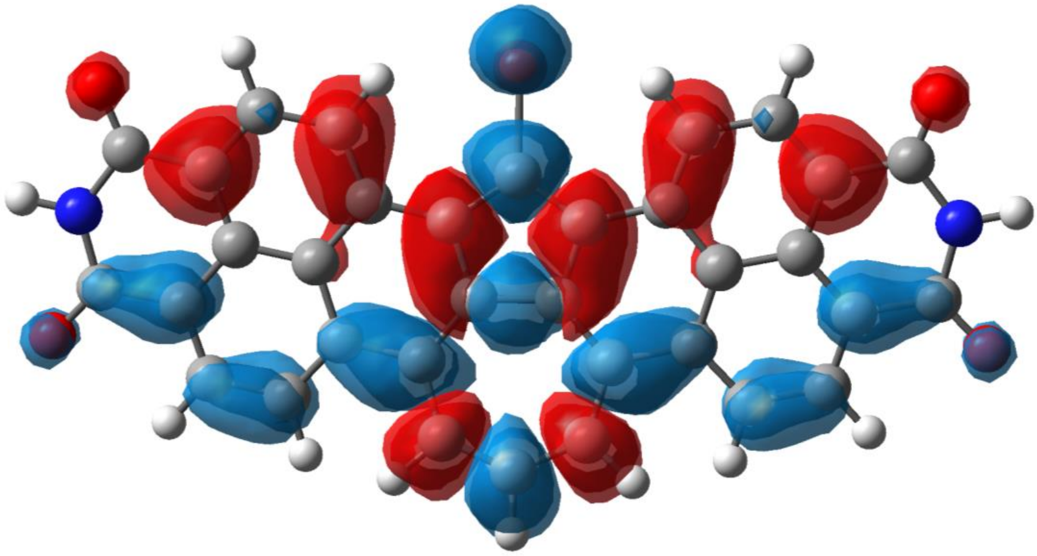

Figure S22. Charge density difference for $S_0 \rightarrow S_1$ transition of compound **5**; Δ*r* = 0.556 Å.

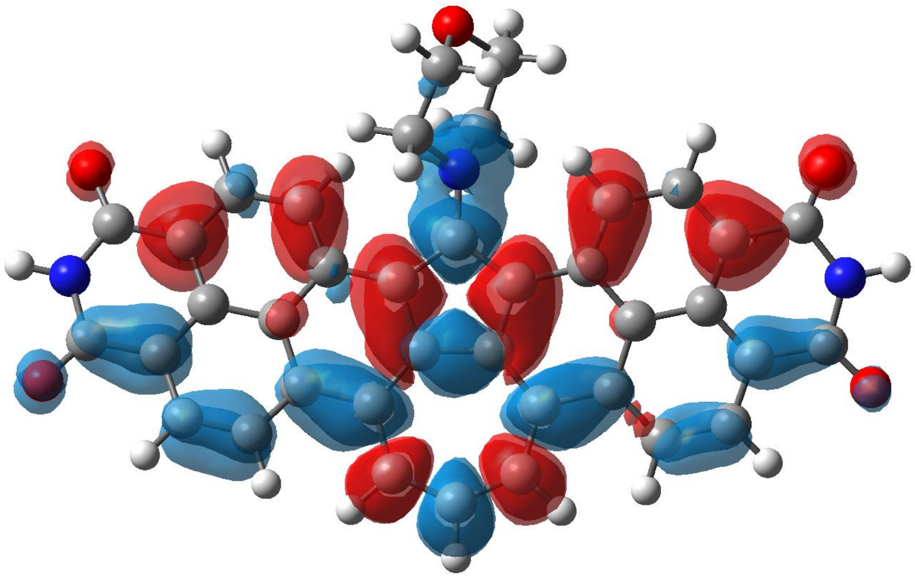

Figure S23. Charge density difference for $S_0 \rightarrow S_1$ transition of compound **6**; Δr = 0.959 Å.

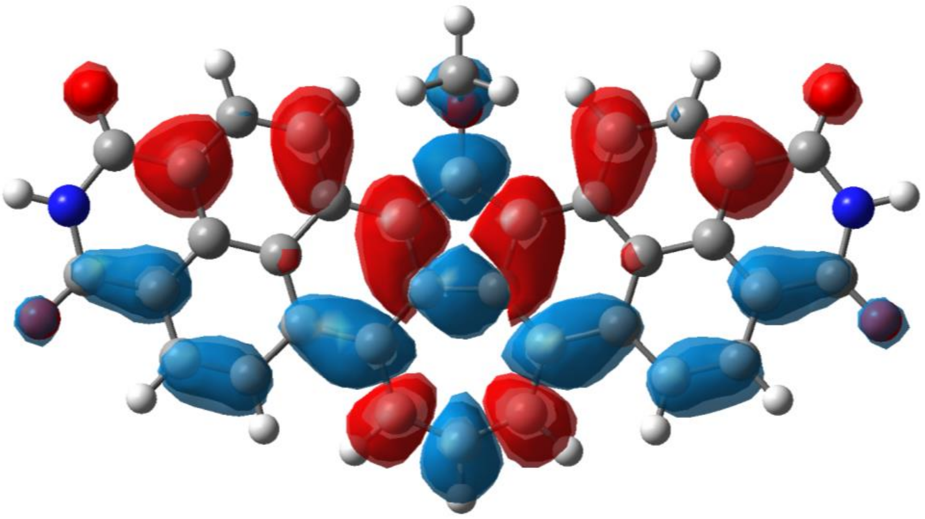

Figure S24. Charge density difference for $S_0 \rightarrow S_1$ transition of compound **7**; Δr = 0.556 Å.

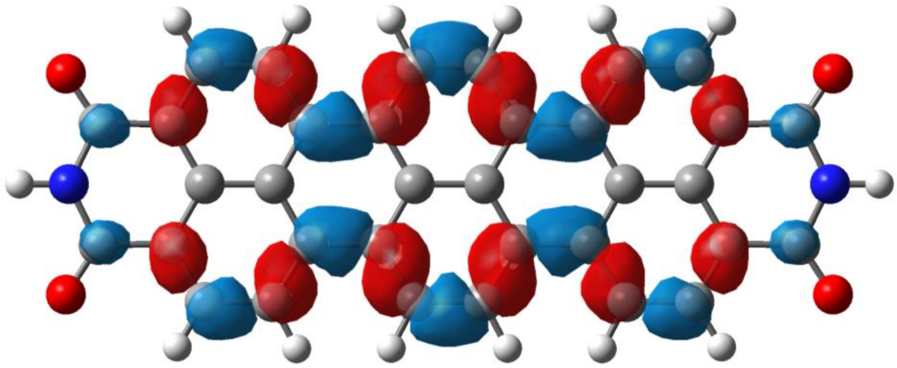

Figure S25. Charge density difference for $S_0 \rightarrow S_1$ transition of **TBI**; Δ*r* = 0.000 Å.

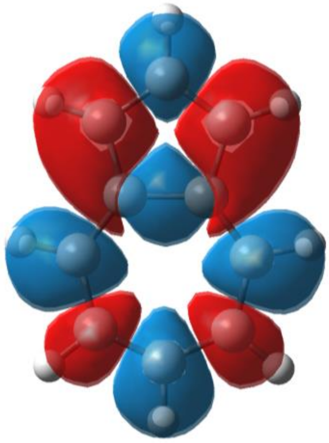

Figure S26. Charge density difference for $S_0 \rightarrow S_1$ transition of azulene; Δ*r* = 0.834 Å.

NICS calculations

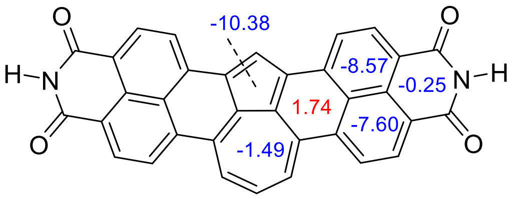

Figure S27. NICS(1) values of compound **1** (1 Å above molecular plane) calculated at B3LYP/6-31+G(d,p) level of theory. Imide substituents replaced with hydrogen atoms for simplicity.

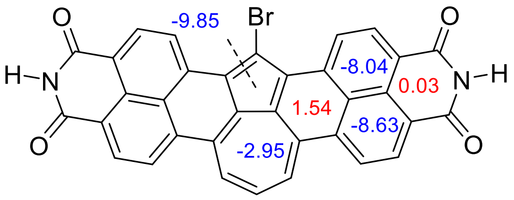

Figure S28. NICS(1) values of compound **5** (1 Å above molecular plane) calculated at B3LYP/6-31+G(d,p) level of therory. Imide substituents replaced with hydrogen atoms for simplicity.

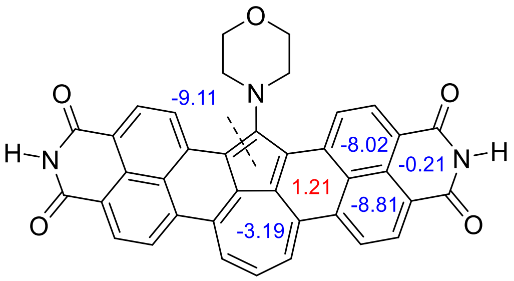

Figure S29. NICS(1) values of compound **6** (1 Å above molecule plane) calculated at B3LYP/6-31+G(d,p) level of theory. Imide substituents replaced with hydrogen atoms for simplicity.

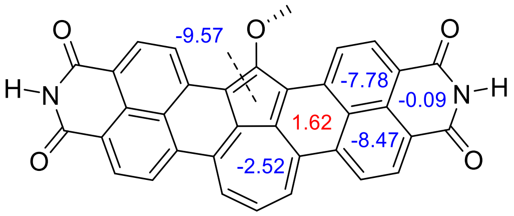

Figure S30. NICS(1) values of compound **7** (1 Å above molecular plane, opposite site of the molecule than OMe group) calculated at B3LYP/6-31+G(d,p) level of theory. Imide substituents replaced with hydrogen atoms for simplicity.

Cartesian coordinates of the ground state optimized structures

Compound **1**
Total energy of the optimized structure: –1714.59080775 a.u.
Number of imaginary frequencies: 0

C	-3.13110000	0.00040000	-1.27560000
C	-3.69160000	0.00050000	0.00000000
C	-3.13110000	0.00040000	1.27560000
C	-1.77550000	0.00010000	1.66240000
C	-0.71710000	0.00010000	0.73950000
C	-0.71710000	0.00010000	-0.73950000
C	-1.77550000	0.00010000	-1.66240000
C	0.64090000	0.00010000	1.14790000
C	1.45220000	0.00020000	0.00000000
C	0.64090000	0.00010000	-1.14790000
C	1.01620000	0.00010000	-2.54090000
C	-1.42000000	-0.00010000	-3.10930000
C	-0.04080000	0.00000000	-3.50850000
C	-1.42000000	-0.00010000	3.10930000
C	-0.04080000	0.00000000	3.50850000
C	1.01620000	0.00010000	2.54090000
C	2.34300000	0.00020000	-2.97360000
C	2.66350000	0.00010000	-4.33400000
C	1.66370000	0.00000000	-5.29380000
C	0.29790000	0.00000000	-4.89560000
C	-0.73380000	-0.00020000	-5.87020000
C	-2.05750000	-0.00040000	-5.47210000
C	-2.39190000	-0.00040000	-4.11320000
C	0.29790000	0.00000000	4.89560000
C	1.66370000	0.00000000	5.29380000
C	2.66350000	0.00010000	4.33400000
C	2.34300000	0.00020000	2.97360000
C	-2.39190000	-0.00040000	4.11320000
C	-2.05750000	-0.00040000	5.47210000
C	-0.73380000	-0.00020000	5.87020000
C	-0.40870000	-0.00010000	7.31880000
N	0.95050000	-0.00010000	7.61500000
C	2.03000000	-0.00010000	6.72540000
C	2.03000000	-0.00010000	-6.72540000
N	0.95050000	-0.00010000	-7.61500000
C	-0.40870000	-0.00010000	-7.31880000
O	-1.24910000	-0.00010000	8.20610000
O	3.17730000	-0.00020000	7.14650000
O	-1.24910000	-0.00010000	-8.20610000
O	3.17730000	-0.00020000	-7.14650000
H	-3.86800000	0.00060000	-2.06840000
H	-4.78020000	0.00080000	0.00000000
H	-3.86800000	0.00060000	2.06840000
H	2.53550000	0.00040000	0.00000000
H	3.14470000	0.00020000	-2.24190000
H	3.69730000	0.00010000	-4.66420000
H	-2.83030000	-0.00060000	-6.23370000
H	-3.44580000	-0.00070000	-3.86640000
H	3.69730000	0.00010000	4.66420000
H	3.14470000	0.00020000	2.24190000
H	-3.44580000	-0.00070000	3.86640000
H	-2.83030000	-0.00060000	6.23370000
H	1.19150000	-0.00010000	8.60110000
H	1.19150000	-0.00010000	-8.60110000

Compound **5**
Total energy of the optimized structure: –4285.68889692 a.u.
Number of imaginary frequencies: 0

C	3.39240800	0.48164000	1.26680400
C	3.95055600	0.61781000	0.00000000
C	3.39240800	0.48164000	-1.26680400
C	2.05811900	0.22377800	-1.63912800
C	0.97824400	0.20557100	-0.73679900
C	0.97824400	0.20557100	0.73679900
C	2.05811900	0.22377800	1.63912800
C	-0.37175800	0.13979900	-1.17143000
C	-1.16783800	0.08557000	0.00000000
C	-0.37175800	0.13979900	1.17143000
C	-0.70336600	0.16518100	2.58862200
C	1.74918600	0.01781300	3.07238800
C	0.38944600	0.05249300	3.51709400
C	1.74918600	0.01781300	-3.07238800
C	0.38944600	0.05249300	-3.51709400
C	-0.70336600	0.16518100	-2.58862200
C	-1.99588600	0.29280700	3.10563400
C	-2.24826400	0.25435800	4.48168400
C	-1.22110200	0.07325900	5.38859000
C	0.11570800	-0.03235200	4.91786300
C	1.18060800	-0.22538900	5.83610400
C	2.47985500	-0.33989000	5.37682200
C	2.75716800	-0.21748300	4.01252200
C	0.11570800	-0.03235200	-4.91786300
C	-1.22110200	0.07325900	-5.38859000
C	-2.24826400	0.25435800	-4.48168400
C	-1.99588600	0.29280700	-3.10563400
C	2.75716800	-0.21748300	-4.01252200
C	2.47985500	-0.33989000	-5.37682200
C	1.18060800	-0.22538900	-5.83610400
C	0.91760600	-0.32447500	-7.29464100
N	-0.41738900	-0.19831300	-7.66294900
C	-1.52519600	-0.00225100	-6.83363600
C	-1.52519600	-0.00225100	6.83363600
N	-0.41738900	-0.19831300	7.66294900
C	0.91760600	-0.32447500	7.29464100
O	1.78967100	-0.50150400	-8.13228300
O	-2.64632500	0.09355000	-7.30963900
O	1.78967100	-0.50150400	8.13228300
O	-2.64632500	0.09355000	7.30963900
H	4.09670400	0.62715100	2.07647700
H	5.01365800	0.85063800	0.00000000
H	4.09670400	0.62715100	-2.07647700
H	-2.83442400	0.42327400	2.43967200
H	-3.26158800	0.35347300	4.85736500
H	3.27393000	-0.51747200	6.09439500
H	3.78701000	-0.32918300	3.69644400
H	-3.26158800	0.35347300	-4.85736500
H	-2.83442400	0.42327400	-2.43967200
H	3.78701000	-0.32918300	-3.69644400
H	3.27393000	-0.51747200	-6.09439500
H	-0.61347300	-0.25857700	-8.65711700
H	-0.61347300	-0.25857700	8.65711700
Br	-3.04256200	-0.15836900	0.00000000

Compound **6**
Total energy of the optimized structure: –2001.17483167 a.u.
Number of imaginary frequencies: 0

C	1.24605400	-3.70664500	0.07109900
C	-0.02439700	-4.27208600	0.16297300
C	-1.28844100	-3.69434800	0.20395700
C	-1.65580700	-2.33315200	0.19705300
C	-0.74520900	-1.27078700	0.06197200
C	0.72848400	-1.27424100	0.00614400
C	1.62847600	-2.35709500	-0.04337900
C	-1.16411400	0.07994300	-0.02290600
C	-0.00440400	0.91298500	0.02887100
C	1.15712000	0.07242700	0.02820100
C	2.56592900	0.39289400	0.15063900
C	3.06582700	-2.03208400	-0.20528400
C	3.50445200	-0.68079100	-0.03042700
C	-3.09009200	-1.98010700	0.32741800
C	-3.51389300	-0.65322100	-0.00086900
C	-2.56013500	0.38597600	-0.26960600
C	3.06224700	1.66905500	0.43288100
C	4.43530900	1.93000100	0.48098000
C	5.35758200	0.93087700	0.22016800
C	4.90375300	-0.39172000	-0.03995800
C	5.83455400	-1.42966500	-0.30552300
C	5.38512400	-2.71346700	-0.56045300
C	4.02013800	-3.00908500	-0.50695900
C	-4.90888700	-0.36211700	-0.08363700
C	-5.33980400	0.91129600	-0.54754000
C	-4.39916600	1.85016600	-0.93610600
C	-3.03026300	1.59215600	-0.79849300
C	-4.05883300	-2.90969100	0.71801500
C	-5.42219500	-2.59721500	0.71151100
C	-5.85481800	-1.35305200	0.28610600
C	-7.30929800	-1.06665900	0.22451400
N	-7.63955600	0.20242000	-0.24268800
C	-6.77899300	1.22702000	-0.64928600
C	6.80019500	1.24625900	0.22560000
N	7.64360000	0.16623400	-0.05186100
C	7.29182500	-1.15200000	-0.32597400
O	-8.17706100	-1.86374800	0.54995300
O	-7.22861100	2.29032200	-1.05121300
O	8.14467500	-1.99590100	-0.56089500
O	7.26662000	2.35406700	0.44979400
H	2.04987200	-4.43221300	0.10060700
H	-0.02680300	-5.35938600	0.21093900
H	-2.10005500	-4.41110200	0.24077400
H	2.36240300	2.46440400	0.64206600
H	4.80277700	2.92630100	0.70646000
H	6.11359800	-3.48433100	-0.78934600
H	3.71667800	-4.02663900	-0.72081200
H	-4.74674200	2.79692400	-1.33728700
H	-2.32405100	2.34049900	-1.13305100
H	-3.76945100	-3.90146400	1.04413700
H	-6.16263600	-3.32993900	1.01515700
H	-8.63141200	0.41067300	-0.29945400
H	8.63760600	0.37169500	-0.05711800
C	0.45353100	3.12508500	-0.98533800
C	-0.87824400	2.97321400	1.09915700
C	0.88657200	4.52099400	-0.53023900
H	-0.37020100	3.22825800	-1.71129700

C	-0.30489700	4.34069000	1.46852400
H	-1.88593700	3.12624800	0.69029700
H	1.85038200	4.47078200	0.00134000
H	0.63777100	4.22459000	2.02820000
N	0.03092000	2.30690600	0.16117200
O	-0.08948900	5.12622400	0.30395200
H	1.28066300	2.62329200	-1.49740400
H	1.00790900	5.17497700	-1.39871100
H	-1.01707000	4.89117900	2.09008900
H	-0.97080600	2.34775600	1.99247500

Compound **7**
Total energy of the optimized structure: –1829.10987689 a.u.
Number of imaginary frequencies: 0

C	-1.27254600	3.27892000	-0.22240900
C	0.00004300	3.84133100	-0.28824200
C	1.27259800	3.27889900	-0.22271200
C	1.65489700	1.92768800	-0.10171000
C	0.74000500	0.86144400	-0.11452800
C	-0.73995000	0.86145100	-0.11445400
C	-1.65483700	1.92772200	-0.10143400
C	1.16093300	-0.48955500	-0.11010300
C	0.00002800	-1.29825500	-0.09381800
C	-1.16089100	-0.48950900	-0.11005500
C	-2.55760700	-0.85530000	-0.15170900
C	-3.09772400	1.58324800	0.00711900
C	-3.51242100	0.21152000	-0.05950300
C	3.09780300	1.58321500	0.00667300
C	3.51248300	0.21145500	-0.05961000
C	2.55766300	-0.85537500	-0.15151300
C	-3.01165000	-2.17164100	-0.27217400
C	-4.37857700	-2.46845800	-0.26788100
C	-5.32290100	-1.46397000	-0.13294800
C	-4.90378500	-0.10877400	-0.02608900
C	-5.86050200	0.92950500	0.11834800
C	-5.44196700	2.24245800	0.23442000
C	-4.08103400	2.56156200	0.17990200
C	4.90384200	-0.10883300	-0.02611300
C	5.32295500	-1.46406700	-0.13243700
C	4.37862100	-2.46861300	-0.26695300
C	3.01168900	-2.17178700	-0.27138100
C	4.08113800	2.56157000	0.17904800
C	5.44208000	2.24246700	0.23355900
C	5.86058500	0.92948100	0.11796100
C	7.31192000	0.62169300	0.16024200
N	7.62993800	-0.72731500	0.04103700
C	6.75885600	-1.81190100	-0.10356100
C	-6.75880100	-1.81178600	-0.10417200
N	-7.62985500	-0.72724200	0.04089900
C	-7.31183000	0.62172200	0.16053300
O	8.18517700	1.46734700	0.28856200
O	7.19804400	-2.94893400	-0.19465700
O	-8.18506600	1.46733400	0.28925500
O	-7.19800100	-2.94877400	-0.19573100
H	-2.07293200	4.00390300	-0.30232400
H	0.00004600	4.92360600	-0.40384100
H	2.07297400	4.00386500	-0.30281700
H	-2.28927600	-2.96985000	-0.38687000
H	-4.72435100	-3.49287600	-0.36275700

H	-6.18976200	3.01783800	0.36434800
H	-3.80928500	3.60386300	0.29120400
H	4.72438600	-3.49307300	-0.36138600
H	2.28931700	-2.97001200	-0.38573500
H	3.80943300	3.60392900	0.29003700
H	6.18988900	3.01789900	0.36318500
H	8.61871300	-0.95559300	0.06531500
H	-8.61863600	-0.95552000	0.06511700
O	0.00009800	-2.66275800	-0.07226000
C	-0.00100300	-3.23441400	1.25005000
H	0.89446400	-2.93192400	1.80408800
H	-0.89722400	-2.93173300	1.80276700
H	-0.00102100	-4.31684500	1.11068500

Terrylene bisimide
Total energy of the optimized structure: –1714.64282596 a.u.
Number of imaginary frequencies: 0

C	5.74115500	-1.23122900	-0.00035800
C	5.03500100	0.00000900	-0.00022400
C	3.60428100	-0.00004400	-0.00004200
C	2.90131700	-1.24572700	0.00006500
C	3.64414400	-2.43091700	-0.00022800
C	5.04107500	-2.42580700	-0.00040300
C	5.74107700	1.23128700	-0.00032300
C	5.04096100	2.42581000	-0.00022800
C	3.64403000	2.43084600	-0.00011900
C	2.90127400	1.24559800	-0.00006100
C	1.43368600	1.24006700	0.00004400
C	0.71947600	-0.00008300	0.00015500
C	1.43369200	-1.24022700	0.00042700
C	0.69540100	2.42257700	0.00011100
C	-0.69527400	2.42260500	0.00000400
C	-1.43365000	1.24007900	-0.00011200
C	-0.71946500	-0.00008100	0.00012400
C	-1.43369000	-1.24022500	0.00056600
C	-0.69535300	-2.42277400	0.00132700
C	0.69535600	-2.42277600	0.00123000
C	-2.90127200	1.24561400	-0.00013000
C	-3.60428200	-0.00001500	0.00015300
C	-2.90132900	-1.24571500	0.00038800
C	7.22195700	-1.25412400	-0.00047300
N	7.83174900	0.00009900	-0.00057300
C	7.22187800	1.25428100	-0.00063200
C	-3.64404500	2.43084800	-0.00053400
C	-5.04095900	2.42581200	-0.00054100
C	-5.74109000	1.23128300	-0.00021500
C	-5.03501400	0.00002300	0.00009200
C	-5.74116300	-1.23121900	0.00032100
C	-5.04111700	-2.42576800	0.00053500
C	-3.64417900	-2.43089500	0.00054300
C	-7.22188800	1.25428000	-0.00019400
N	-7.83176000	0.00009700	0.00010100
C	-7.22196700	-1.25412100	0.00024400
O	7.89270400	-2.27585600	-0.00062900
O	7.89255100	2.27606100	-0.00049400
O	-7.89256700	2.27605800	-0.00054200
O	-7.89270200	-2.27586100	0.00071100
H	3.14338400	-3.39141100	-0.00033100
H	5.60059500	-3.35557600	-0.00048400

H	5.60045200	3.35559500	-0.00047000
H	3.14318800	3.39130200	-0.00018500
H	1.19707300	3.38267700	0.00022100
H	-1.19687500	3.38273500	0.00007200
H	-1.19698800	-3.38288700	0.00215100
H	1.19700000	-3.38288500	0.00197000
H	8.84686800	0.00012300	-0.00072300
H	-3.14321800	3.39130600	-0.00085100
H	-5.60045300	3.35559600	-0.00074700
H	-5.60065400	-3.35552600	0.00053600
H	-3.14344800	-3.39140300	0.00056800
H	-8.84687900	0.00013300	0.00014400

Azulene
Total energy of the optimized structure: –385.83815971 a.u.
Number of imaginary frequencies: 0

C	2.50396300	0.00004400	-0.00002500
C	1.91103600	-1.26612800	-0.00009400
C	0.55205500	-1.59448300	-0.00009100
C	1.91101900	1.26613900	0.00006200
C	-0.55456300	-0.75021600	-0.00003300
C	0.55199000	1.59449500	0.00010300
C	-0.55462100	0.75022700	0.00006800
H	3.59307400	0.00002600	-0.00004000
H	2.59910100	-2.10874500	-0.00011800
H	0.32403700	-2.66052600	-0.00003200
H	2.59899100	2.10883600	0.00007000
H	0.32404400	2.66055200	0.00008900
C	-1.90194900	-1.14997500	0.00028500
H	-2.24865400	-2.17672200	0.00053100
C	-2.70824300	-0.00003300	-0.00018400
H	-3.79333400	-0.00007500	-0.00025100
C	-1.90208800	1.14993100	-0.00010300
H	-2.24885200	2.17665400	-0.00018000

6. NMR Spectra

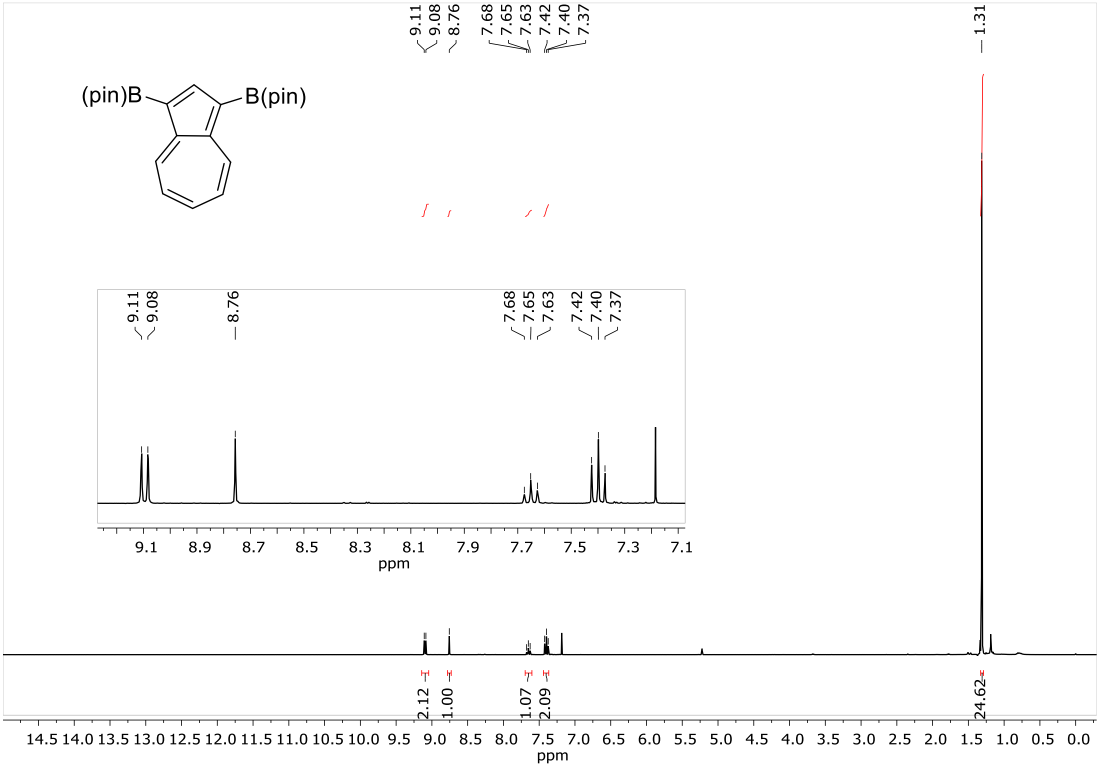

Figure S31. ^{1}H NMR spectrum of **3** (400 MHz, $CDCl_3$, 298 K).

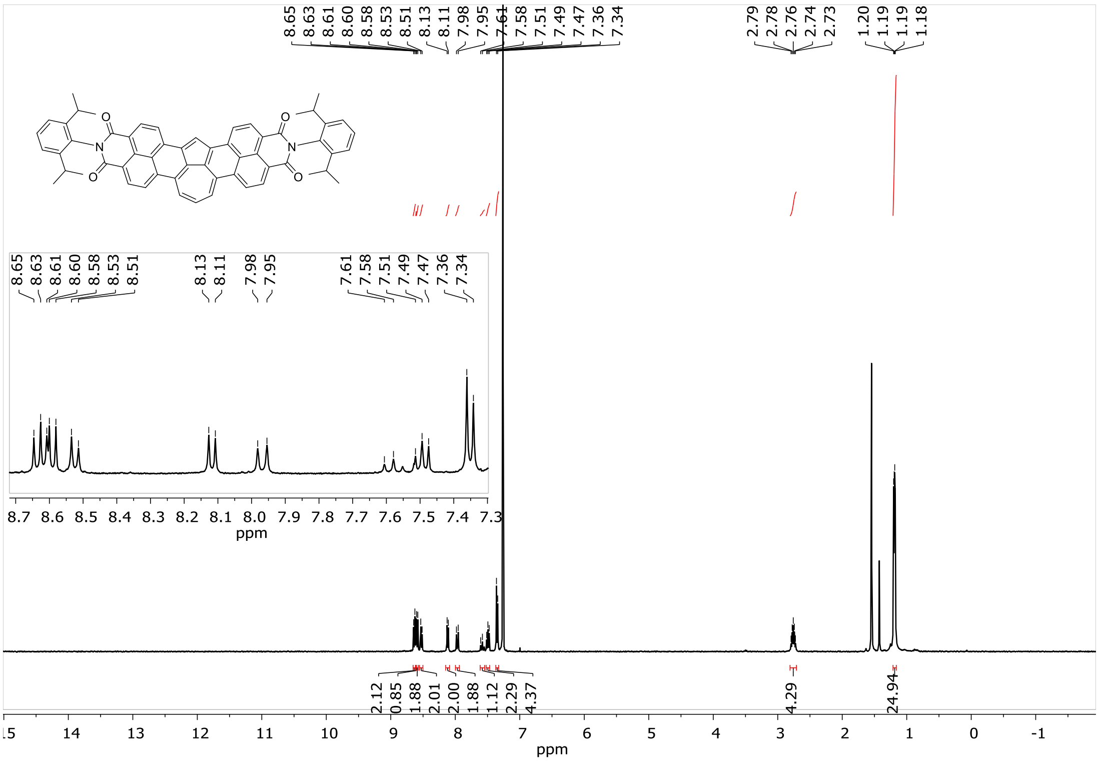

Figure S32. ^{1}H NMR spectrum of **1** (400 MHz, $CDCl_3$, 298 K).

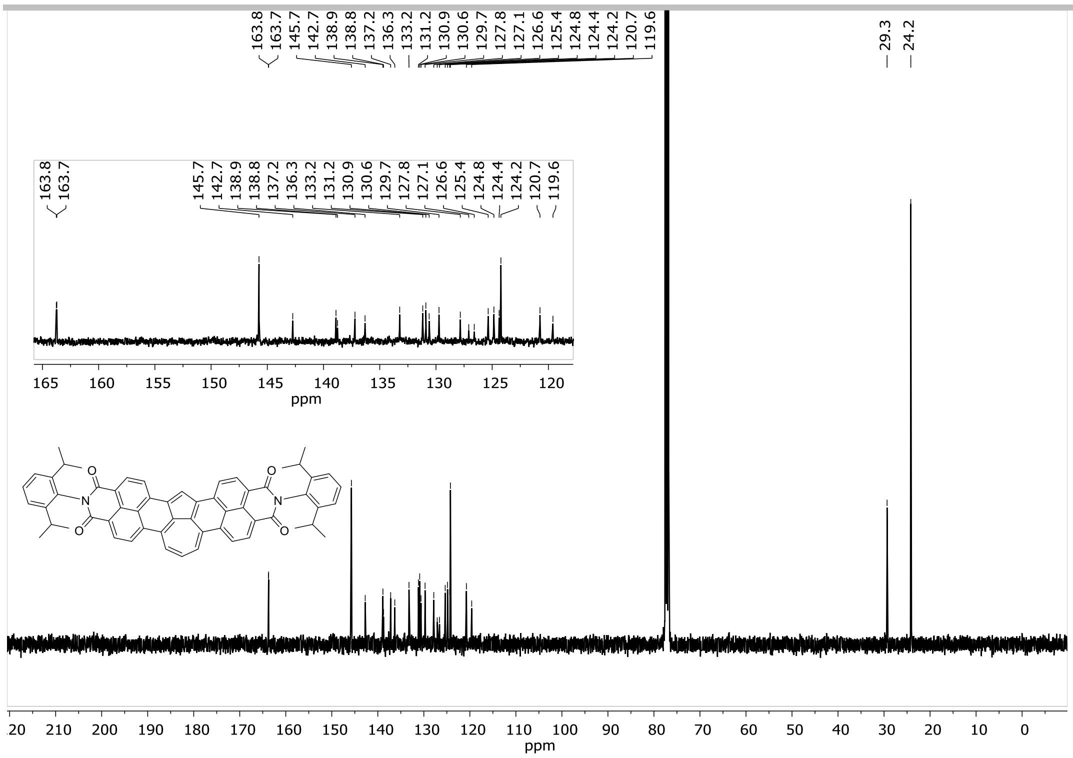

Figure S33. ^{13}C NMR spectrum of **1** (101 MHz, $CDCl_3$, 298 K).

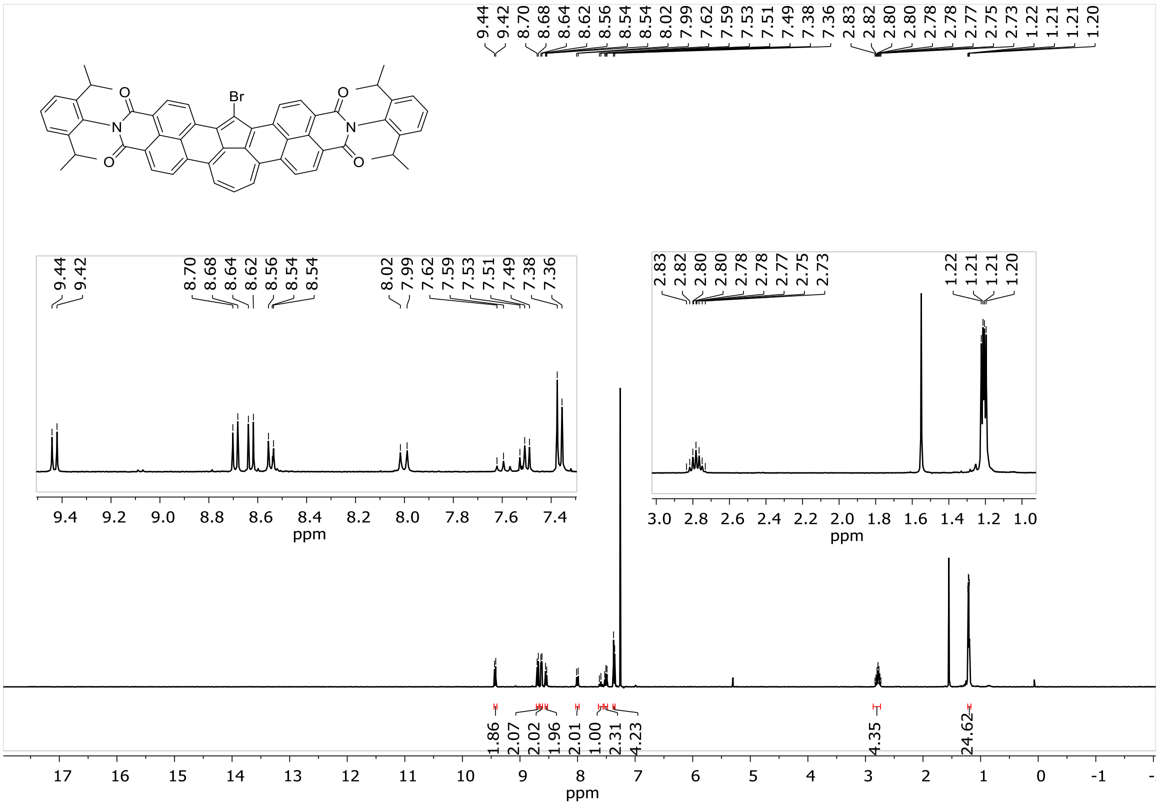

Figure S34. ^{1}H NMR spectrum of **5** (400 MHz, $CDCl_3$, 298 K).

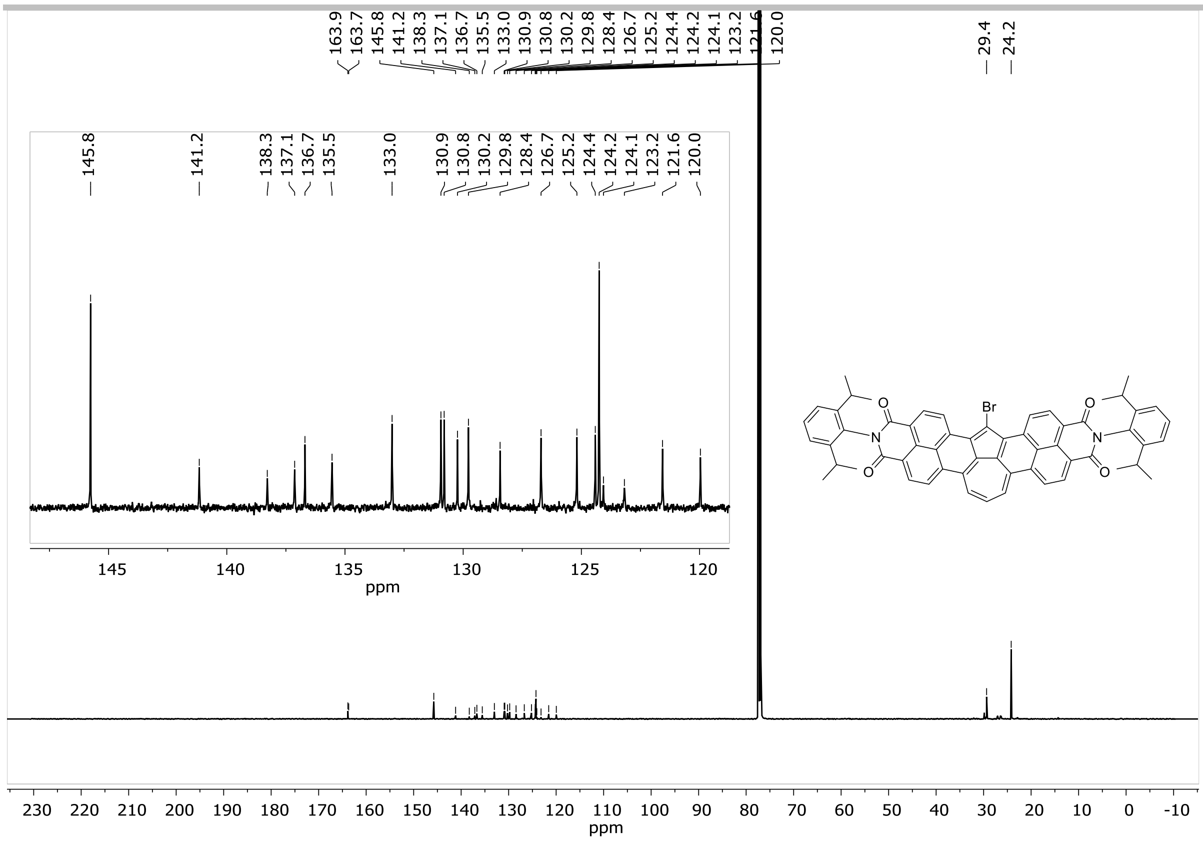

Figure S35. ^{13}C NMR spectrum of **5** (151 MHz, $CDCl_3$, 298 K).

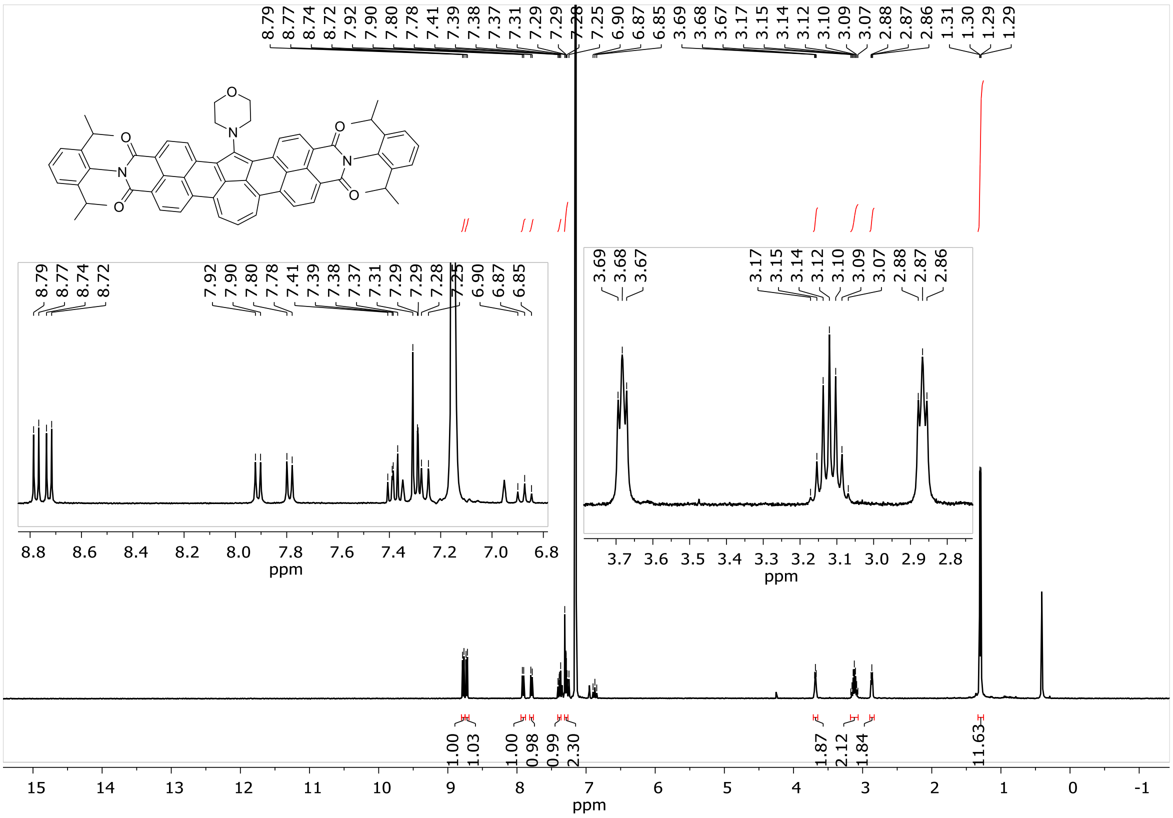

Figure S36. ^{1}H NMR spectrum of **6** (400 MHz, C_6D_6, 298 K).

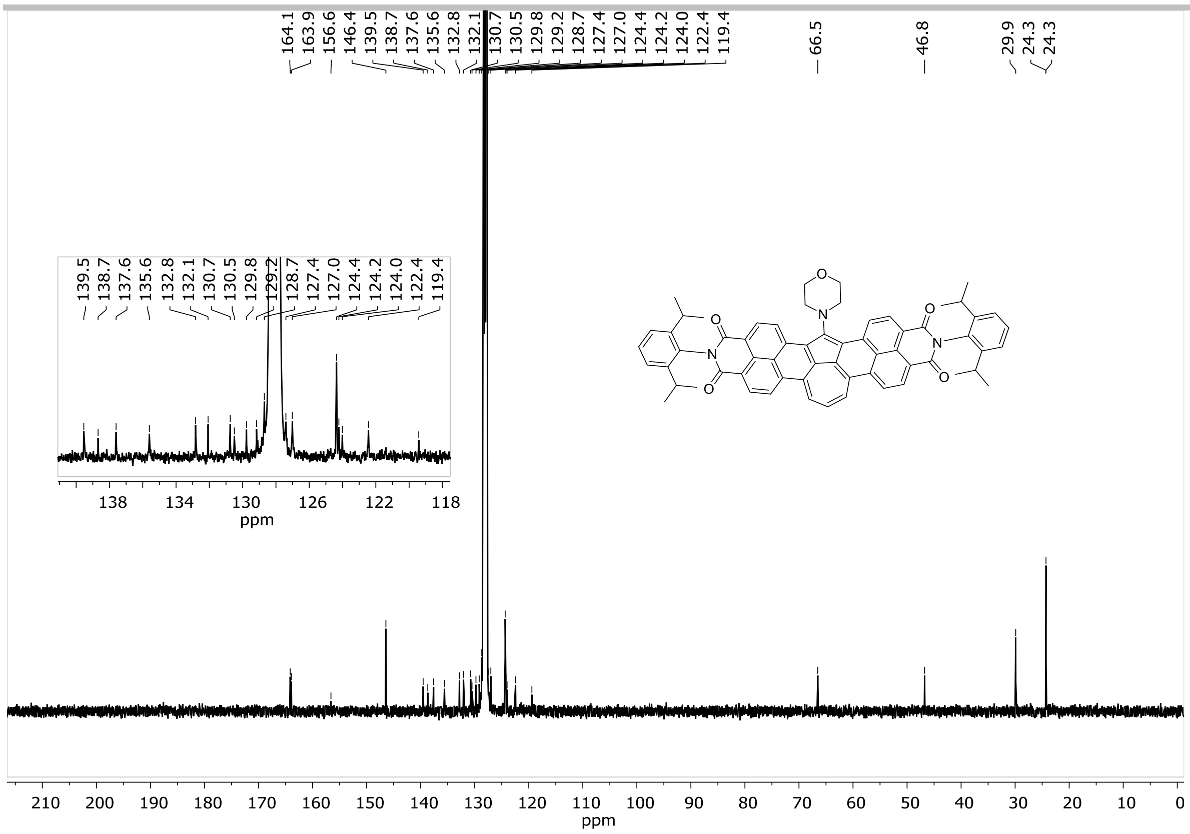

Figure S37. ^{13}C NMR spectrum of **6** (101 MHz, C_6D_6, 298 K).

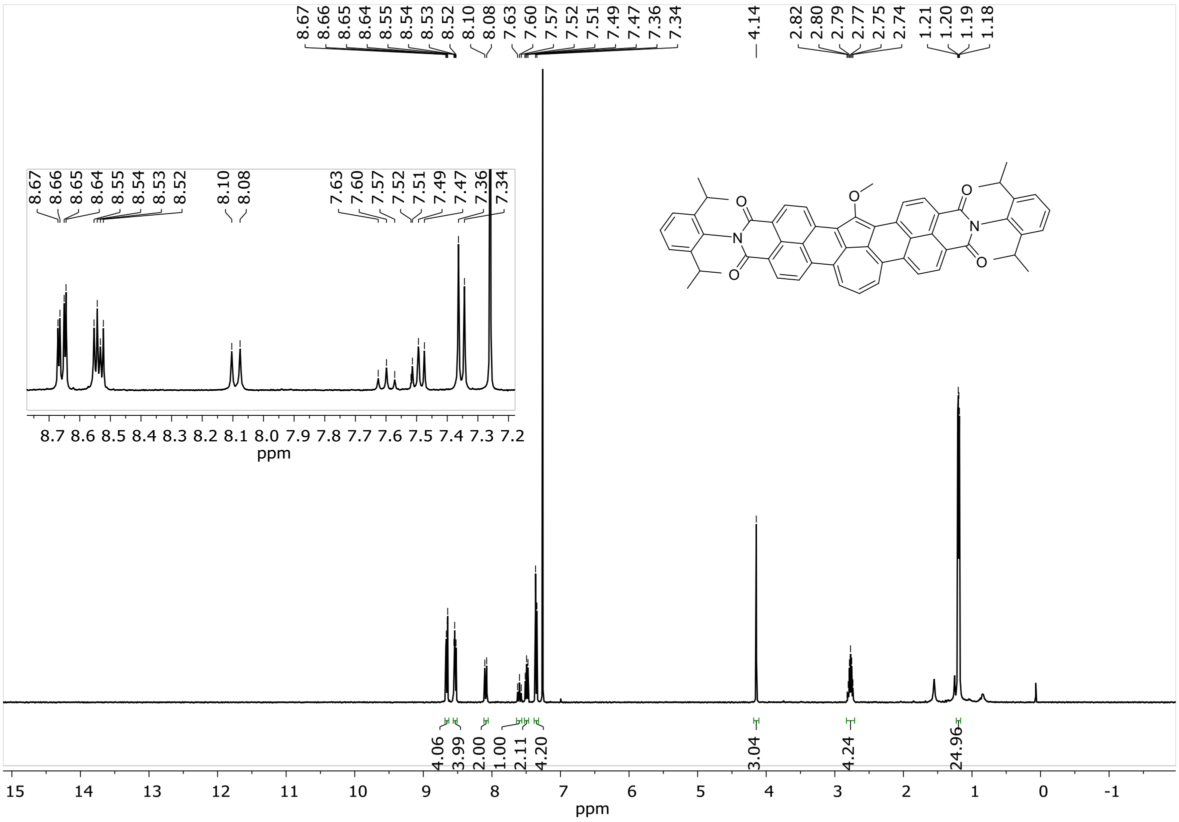

Figure S38. ^{1}H NMR spectrum of **7** (400 MHz, $CDCl_3$, 298 K).

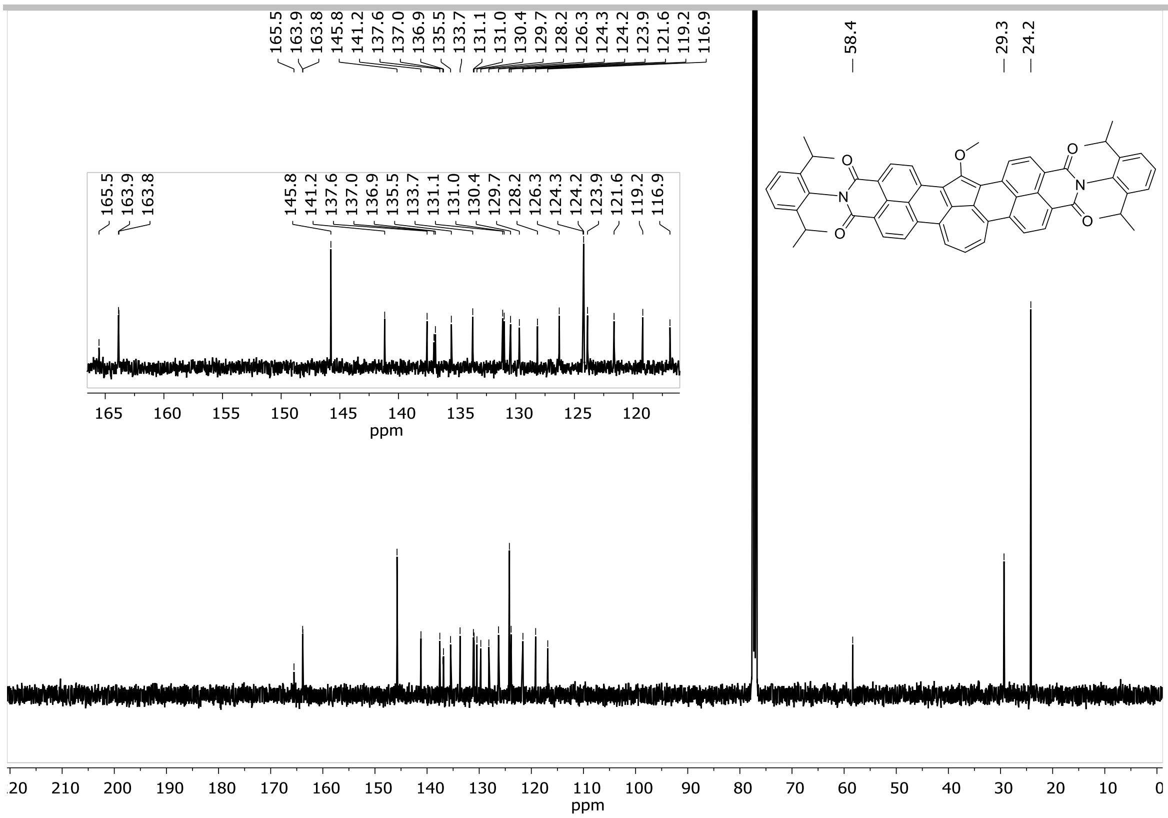

Figure S39. ^{13}C NMR spectrum of **7** (101 MHz, $CDCl_3$, 298 K).

7. Mass Spectra

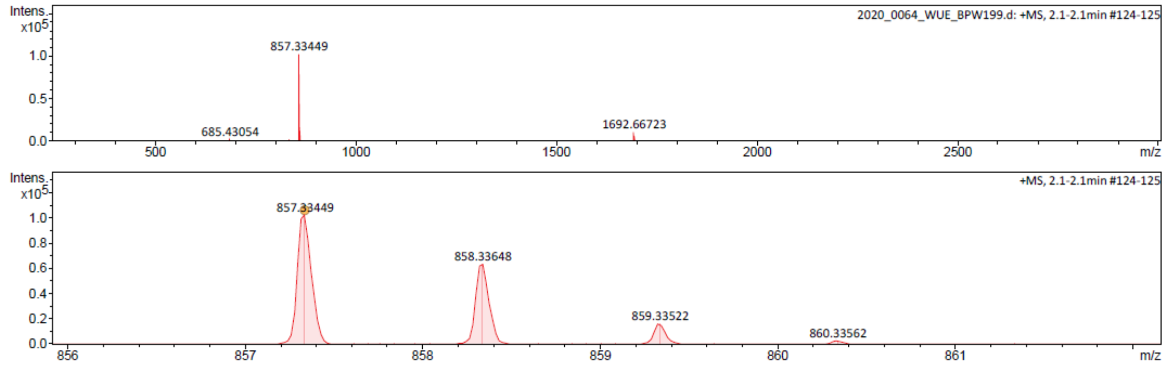

Meas. m/z	#	Ion Formula	m/z	err [ppm]	mSigma	# mSigma	Score	rdb	e⁻ Conf	N-Rule
857.33449	1	C58H46N2NaO4	857.33498	0.57	31.3	1	100.00	36.5	even	ok

Figure S40. HRMS spectrum (ESI-TOF) of compound **1**.

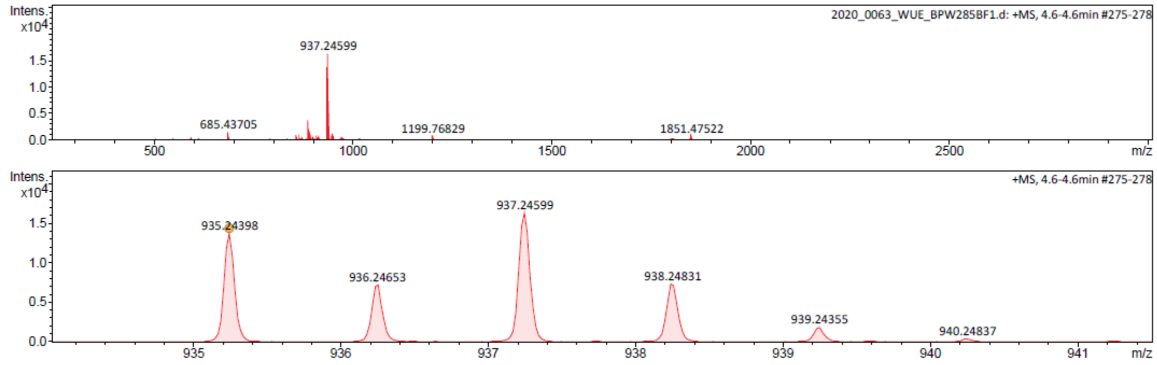

Meas. m/z	#	Ion Formula	m/z	err [ppm]	mSigma	# mSigma	Score	rdb	e⁻ Conf	N-Rule
935.24398	1	C58H45BrN2NaO4	935.24549	1.62	67.5	1	100.00	36.5	even	ok

Figure S41. HRMS spectrum (ESI-TOF) of compound 5.

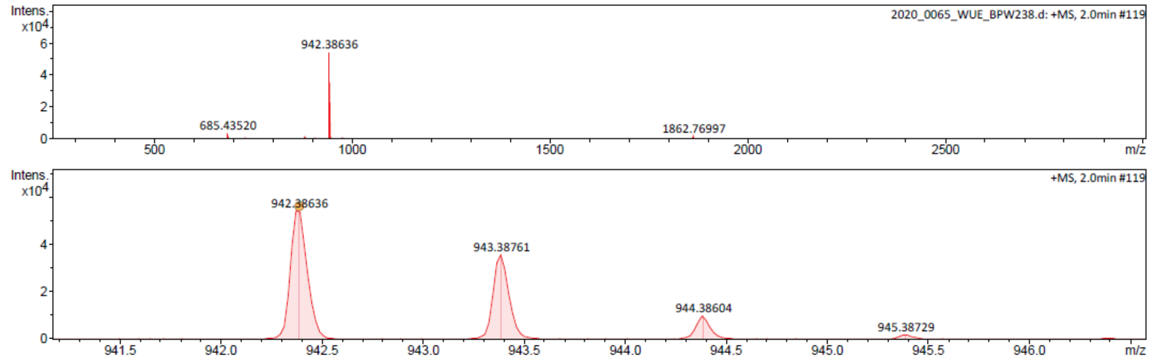

Meas. m/z	#	Ion Formula	m/z	err [ppm]	mSigma	# mSigma	Score	rdb	e⁻ Conf	N-Rule
942.38636	1	C62H53N3NaO5	942.38774	1.46	35.3	1	100.00	37.5	even	ok

Figure S42. HRMS spectrum (ESI-TOF) of compound **6**.

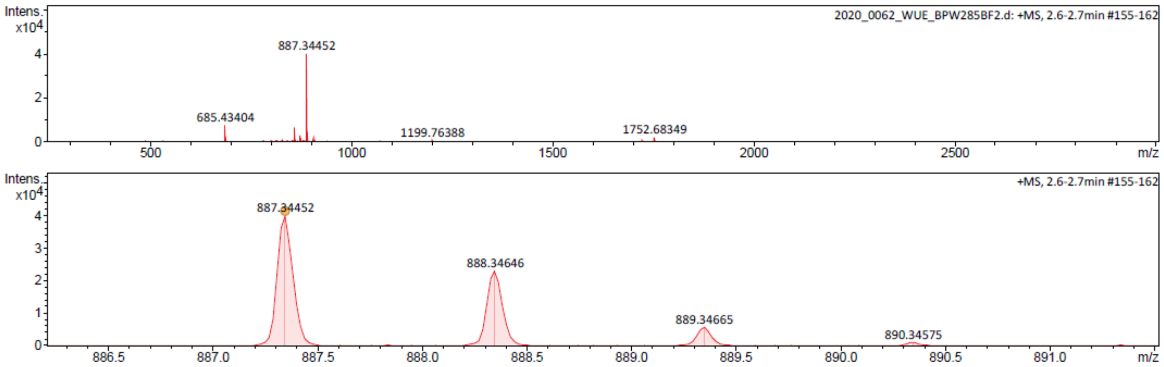

Meas. m/z	#	Ion Formula	m/z	err [ppm]	mSigma	# mSigma	Score	rdb	e¯ Conf	N-Rule
887.34452	1	C59H48N2NaO5	887.34554	1.15	51.1	1	100.00	36.5	even	ok

Figure S43. HRMS spectrum (ESI-TOF) of compound **7**.

8. References

[1] M. J. Frisch, G. W. Trucks, H. B. Schlegel, G. E. Scuseria, M. A. Robb, J. R. Cheeseman, G. Scalmani, V. Barone, G. A. Petersson, H. Nakatsuji, X. Li, M. Caricato, A. V. Marenich, J. Bloino, B. G. Janesko, R. Gomperts, B. Mennucci, H. P. Hratchian, J. V. Ortiz, A. F. Izmaylov, J. L. Sonnenberg, Williams, F. Ding, F. Lipparini, F. Egidi, J. Goings, B. Peng, A. Petrone, T. Henderson, D. Ranasinghe, V. G. Zakrzewski, J. Gao, N. Rega, G. Zheng, W. Liang, M. Hada, M. Ehara, K. Toyota, R. Fukuda, J. Hasegawa, M. Ishida, T. Nakajima, Y. Honda, O. Kitao, H. Nakai, T. Vreven, K. Throssell, J. A. Montgomery Jr., J. E. Peralta, F. Ogliaro, M. J. Bearpark, J. J. Heyd, E. N. Brothers, K. N. Kudin, V. N. Staroverov, T. A. Keith, R. Kobayashi, J. Normand, K. Raghavachari, A. P. Rendell, J. C. Burant, S. S. Iyengar, J. Tomasi, M. Cossi, J. M. Millam, M. Klene, C. Adamo, R. Cammi, J. W. Ochterski, R. L. Martin, K. Morokuma, O. Farkas, J. B. Foresman, D. J. Fox, Wallingford, CT, **2016**.

[2] T. Lu, F. Chen, *J. Comput. Chem.* **2012**, *33*, 580-592.

[3] J. Dubovik, A. Bredihhin, *Synthesis* **2015**, *47*, 538-548.

[4] K. Shoyama, D. Schmidt, M. Mahl, F. Würthner, *Org. Lett.* **2017**, *19*, 5328-5331.

[5] S. S. Zalesskiy, V. P. Ananikov, *Organometallics* **2012**, *31*, 2302-2309.

[6] G. M. Sheldrick, *Acta Crystallogr. A* **2008**, *64*, 112-122.

[7] J. Yao, Z. Cai, Z. Liu, C. Yu, H. Luo, Y. Yang, S. Yang, G. Zhang, D. Zhang, *Macromolecules* **2015**, *48*, 2039-2047.

[8] J. Kruszewski, T. M. Krygowski, *Tetrahedron Lett.* **1972**, *13*, 3839-3842.

[9] B. Dittrich, F. P. A. Fabbiani, J. Henn, M. U. Schmidt, P. Macchi, K. Meindl, M. A. Spackman, *Acta Crystallogr. B* **2018**, *74*, 416-426.

[10] R. Regar, K. S. Mehra, R. Bhowal, J. Sankar, *Eur. J. Org. Chem.* **2019**, 6278-6284.

[11] N. G. Connelly, W. E. Geiger, *Chem. Rev.* **1996**, *96*, 877-910.

[12] F. O. Holtrup, G. R. J. Müller, H. Quante, S. De Feyter, F. C. De Schryver, K. Müllen, *Chem. Eur. J.* **1997**, *3*, 219-225.

[13] E. Kloster-Jensen, E. Kováts, A. Eschenmoser, E. Heilbronner, *Helv. Chim. Acta* **1956**, *39*, 1051-1067.
